# A body shape index (ABSI) is associated inversely with post-menopausal progesterone-receptor-negative breast cancer risk in a large European cohort

**DOI:** 10.1186/s12885-023-11056-1

**Published:** 2023-06-19

**Authors:** Sofia Christakoudi, Konstantinos K. Tsilidis, Laure Dossus, Sabina Rinaldi, Elisabete Weiderpass, Christian S. Antoniussen, Christina C. Dahm, Anne Tjønneland, Lene Mellemkjær, Verena Katzke, Rudolf Kaaks, Matthias B. Schulze, Giovanna Masala, Sara Grioni, Salvatore Panico, Rosario Tumino, Carlotta Sacerdote, Anne M. May, Evelyn M. Monninkhof, J. Ramón Quirós, Catalina Bonet, Maria-Jose Sánchez, Pilar Amiano, María-Dolores Chirlaque, Marcela Guevara, Ann H. Rosendahl, Tanja Stocks, Aurora Perez-Cornago, Sandar Tin Tin, Alicia K. Heath, Elom K. Aglago, Laia Peruchet-Noray, Heinz Freisling, Elio Riboli

**Affiliations:** 1 Department of Epidemiology and Biostatistics, School of Public Health, Imperial College London, St Mary’s Campus, Norfolk Place, London, W2 1PG UK; 2 Department of Inflammation Biology, School of Immunology & Microbial Sciences, King’s College London, London, UK; 3 Department of Hygiene and Epidemiology, University of Ioannina School of Medicine, Ioannina, Greece; 4 International Agency for Research on Cancer (IARC/WHO), 25 avenue Tony Garnier, Lyon, CS 90627, 69366 LYON CEDEX 07 France; 5 Department of Public Health, Aarhus University, Bartholins Allé 2, Aarhus C, DK-8000 Denmark; 6 Diet, Cancer and Health, Danish Cancer Society Research Center, Strandboulevarden 49, Copenhagen, DK-2100 Denmark; 7 Department of Public Health, University of Copenhagen, Copenhagen, Denmark; 8 Department of Cancer Epidemiology, German Cancer Research Center (DKFZ), Heidelberg, Germany; 9 Department of Molecular Epidemiology, German Institute of Human Nutrition Potsdam-Rehbruecke, Nuthetal, 14558 Germany; 10 Institute of Nutritional Science, University of Potsdam, Nuthetal, Germany; 11 Institute for Cancer Research, Prevention and Clinical Network (ISPRO), Florence, Italy; 12 Epidemiology and Prevention Unit, Fondazione IRCCS Istituto Nazionale dei Tumori di Milano, Via Venezian 1, Milano, 20133 Italy; 13 Dipartimento di Medicina Clinica e Chirurgia, Federico II University, Naples, Italy; 14 Hyblean Association Epidemiological Research AIRE – ONLUS, Ragusa, Italy; 15 Unit of Cancer Epidemiology, Città della Salute e della Scienza University-Hospital, Via Santena 7, Turin, 10126 Italy; 16 Julius Center for Health Sciences and Primary Care, University Medical Center Utrecht, Utrecht University, P.O. Box 85500, Utrecht, 3508 GA Netherlands; 17 Public Health Directorate, Asturias, Spain; 18 Unit of Nutrition and Cancer, Catalan Institute of Oncology - ICO, L’Hospitalet de Llobregat, Barcelona, Spain; 19 Nutrition and Cancer Group; Epidemiology, Public Health, Cancer Prevention and Palliative Care Program, Bellvitge Biomedical Research Institute - IDIBELL, L’Hospitalet de Llobregat, Barcelona, Spain; 20 Escuela Andaluza de Salud Pública (EASP), Granada, 18011 Spain; 21 Instituto de Investigación Biosanitaria ibs.GRANADA, Granada, 18012 Spain; 22 Centro de Investigación Biomédica en Red de Epidemiología y Salud Pública (CIBERESP), Madrid, 28029 Spain; 23 Department of Preventive Medicine and Public Health, University of Granada, Granada, 18071 Spain; 24 Ministry of Health of the Basque Government, Sub Directorate for Public Health and Addictions of Gipuzkoa, San Sebastian, Spain; 25 Epidemiology of Chronic and Communicable Diseases Group, Biodonostia Health Research Institute, San Sebastián, Spain; 26 Spanish Consortium for Research on Epidemiology and Public Health (CIBERESP), Instituto de Salud Carlos III, Madrid, Spain; 27 Department of Epidemiology, Regional Health Council, IMIB-Arrixaca, Murcia University, Murcia, Spain; 28 CIBER in Epidemiology and Public Health (CIBERESP), Madrid, Spain; 29 Navarra Public Health Institute, Pamplona, Spain; 30 Navarra Institute for Health Research (IdiSNA), Pamplona, Spain; 31 Department of Clinical Sciences Lund, Oncology, Lund University and Skåne University Hospital, Lund, Sweden; 32 Department of Translational Medicine, Lund University, Malmö, Sweden; 33 Cancer Epidemiology Unit, Nuffield Department of Population Health, University of Oxford, Oxford, UK; 34 Department of Clinical Sciences, Faculty of Medicine, University of Barcelona, Barcelona, Spain

**Keywords:** Obesity, Body shape, Waist size, ABSI, Hip size, Breast cancer

## Abstract

**Background:**

Associations of body shape with breast cancer risk, independent of body size, are unclear because waist and hip circumferences are correlated strongly positively with body mass index (BMI).

**Methods:**

We evaluated body shape with the allometric “a body shape index” (ABSI) and hip index (HI), which compare waist and hip circumferences, correspondingly, among individuals with the same weight and height. We examined associations of ABSI, HI, and BMI (per one standard deviation increment) with breast cancer overall, and according to menopausal status at baseline, age at diagnosis, and oestrogen and progesterone receptor status (ER+/-PR+/-) in multivariable Cox proportional hazards models using data from the European Prospective Investigation into Cancer and Nutrition (EPIC) cohort.

**Results:**

During a mean follow-up of 14.0 years, 9011 incident breast cancers were diagnosed among 218,276 women. Although there was little evidence for association of ABSI with breast cancer overall (hazard ratio HR = 0.984; 95% confidence interval: 0.961–1.007), we found borderline inverse associations for post-menopausal women (HR = 0.971; 0.942-1.000; n = 5268 cases) and breast cancers diagnosed at age ≥ 55 years (HR = 0.976; 0.951–1.002; n = 7043) and clear inverse associations for ER + PR- subtypes (HR = 0.894; 0.822–0.971; n = 726) and ER-PR- subtypes (HR = 0.906; 0.835–0.983 n = 759). There were no material associations with HI. BMI was associated strongly positively with breast cancer overall (HR = 1.074; 1.049–1.098), for post-menopausal women (HR = 1.117; 1.085–1.150), for cancers diagnosed at age ≥ 55 years (HR = 1.104; 1.076–1.132), and for ER + PR + subtypes (HR = 1.122; 1.080–1.165; n = 3101), but not for PR- subtypes.

**Conclusions:**

In the EPIC cohort, abdominal obesity evaluated with ABSI was not associated with breast cancer risk overall but was associated inversely with the risk of post-menopausal PR- breast cancer. Our findings require validation in other cohorts and with a larger number of PR- breast cancer cases.

**Supplementary Information:**

The online version contains supplementary material available at 10.1186/s12885-023-11056-1.

## Background

Associations of adult body size with breast cancer risk are reasonably well established. Adult body mass index (BMI) is associated positively with post-menopausal breast cancer but inversely with pre-menopausal breast cancer [1–3]. Associations of body shape with breast cancer risk, however, are less clear because the available knowledge is based on measurements of waist and hip circumferences, which are strongly positively correlated with BMI. Consequently, associations with waist and hip circumferences, when examined individually, reflect associations with BMI and, when adjusted for BMI, show considerably larger confidence intervals, with risk estimates biased upwards for positive associations and downwards for inverse associations [4].

The allometric “a body shape index” (ABSI) and hip index (HI) [5, 6] were created to overcome the limitations of waist and hip circumferences. Similarly to BMI, which reflects relative weight compared to individuals with the same height, ABSI and HI reflect relative waist and hip size, correspondingly, compared to individuals with the same weight and height. While most lean individuals have small waist circumference and most obese individuals have large waist circumference, the proportion of women with large ABSI is comparable for all BMI categories [7]. A similar logic applies to HI. As ABSI and HI are not correlated with BMI, they can all be combined in unbiased joint analyses of body shape and body size [4].

We have previously examined associations of ABSI and HI with breast cancer risk in UK Biobank, but found no material evidence for association [4]. This was unexpected, because glucose and glycated haemoglobin were associated positively with both ABSI and BMI and inversely with HI in UK Biobank [8], and insulin resistance has been associated with breast cancer risk [9]. Therefore, a positive association of ABSI with post-menopausal breast cancer risk would be expected and has been reported for breast cancer overall by a different group using UK Biobank data [10], while an inverse association of ABSI with post-menopausal breast cancer has been reported in a case-control study [11], highlighting inconsistencies between studies. Furthermore, oestrogens, which contribute to breast cancer development [12], are generated in subcutaneous adipose tissue via aromatisation from androgens, with regional differences in aromatase expression and highest levels in the gluteofemoral region [13]. Therefore, a positive association of HI with breast cancer risk is plausible. It is possible, however, that associations of body shape with breast cancer risk differ according to hormone receptor status of the cancer, which would determine the sensitivity of the cancer to hormonal exposures, but information on hormone receptor status was not available in UK Biobank.

Therefore, we used data from the European Prospective Investigation into Cancer and Nutrition (EPIC) cohort to examine prospectively the associations of ABSI and HI with the risk of breast cancer overall, and according to menopausal status at the anthropometric assessment, age at breast cancer diagnosis, and hormone receptor status of the cancer.

## Methods

### Study population

EPIC is a multicentre cohort including participants from ten European countries (recruited in 23 centres from 1991 to 1999). Details regarding the recruitment and the collection of information on socio-demographic, lifestyle, reproductive, and dietary factors has previously been described [14]. In accordance with our previous publications examining associations of obesity with cancer risk [15, 16], we excluded 149,622 women (40.7% of all EPIC women). Among the excluded women, 16,614 were from Greece (excluded due to administrative reasons), 101,849 had missing waist and hip circumference measurements (not collected in Norway and in Umeå, Sweden), and 23,640 had prevalent cancer. The remaining excluded women had either missing weight or height measurements (n = 1899), or extreme anthropometric characteristics (n = 216), or missing lifestyle or dietary questionnaires (n = 709), or extreme energy intake (within the top or bottom 1% of the cohort distribution, n = 4304), or were pregnant at cohort recruitment (n = 391). A more detailed count of all exclusion criteria is presented in Supplementary Fig. S1. Women from eight countries were included in this study: Denmark, France, Germany, Italy, the Netherlands, Spain, Sweden, and the United Kingdom (UK).

### Anthropometric assessment and indices

Anthropometric measurements were obtained within one year of cohort recruitment, except for France, where all available measurements were obtained on average 3.8 years after recruitment (standard deviation 1.3 years), and the UK (Oxford cohort), for which predictive equations based on available measurements were applied to self-reported values [17]. To adjust for clothing worn during the anthropometric assessment, 1.0 kg was removed from weight for light clothing, and 1.5 kg was removed from weight and 2.0 cm from waist and hip circumferences for normal clothing [18].

We calculated ABSI as waist circumference (WC, m) ∗ weight ^− 2/3^ (kg) ∗ height ^5/6^ (m) [5] and additionally multiplied this by 1000. We calculated HI as hip circumference (HC, cm) ∗ weight ^− 0.482^ (kg) ∗ height ^0.310^ (cm) [6] and BMI as weight (kg) * height ^− 2^ (m). For the main analyses, we calculated study-specific z-scores, as value minus mean (72.939 for ABSI; 64.806 for HI; 25.304 for BMI) divided by standard deviation (5.070 for ABSI; 2.717 for HI; 4.464 for BMI). To explore non-linearity, we derived study-specific quintile categories (Q1-Q5).

### Assessment of menopausal status, lifestyle, reproductive, and dietary factors

Women completed detailed questionnaires on lifestyle, menstrual and reproductive history, use of exogenous hormones, and diet at cohort recruitment [14], and this information was harmonised between EPIC centres. Menopausal status at cohort recruitment had previously been defined, taking into account the number of periods per year and hormone use, as pre-menopausal (at least ten periods per year and no current use of hormone replacement therapy (HRT) or oral contraceptives), peri-menopausal (less than ten periods per year), surgically post-menopausal (bilateral oophorectomy), and naturally post-menopausal (no menstruation and no hysterectomy) [3]. Menopausal status for women with hysterectomy, or with missing information for menstruation, or using hormones was determined according to age cut-offs as follows: pre-menopausal, if aged < 46 years at cohort recruitment; peri-menopausal, if aged 46 to < 55 years at cohort recruitment; post-menopausal, if aged ≥ 55 at cohort recruitment. For the current study, we combined natural and surgical post-menopause into one group (post-menopausal). To account for the delay in anthropometric measurements in France, we assigned all women from France aged ≥ 55 years at the anthropometric assessment to post-menopausal.

### Cancer ascertainment and subtypes

Incident cancer cases were identified by record linkage to cancer registries in Denmark, Italy, the Netherlands, Spain, Sweden, and the UK, and using a combination of active follow-up of study participants, cancer and pathology registries, and health insurance records in France and Germany [14]. First primary breast cancer was defined with code C50 according to the International Classification of Diseases for Oncology (ICD-O). The following were censored at the date of diagnosis: breast cancer with rare morphology (codes 8801, 8804, 8810, 8980, 8982, 9020, 9120, 9590, 9675, 9690, 9691), breast cancer with behavioural code other than 3 (malignant, primary site), or a first primary cancer in another location, defined as in our previous study on weight change and risk of cancer [15]. Breast cancer cases were divided into two groups according to age at diagnosis: <55 years and ≥ 55 years, as a proxy of menopausal status at diagnosis.

Oestrogen receptor (ER) and progesterone receptor (PR) status were available for 52.1% of breast cancers included in this study and human epidermal receptor 2 (HER2) status was available for 32.8%, but not for women from Denmark. Positive hormone receptor status was determined using one of the following standardised thresholds: ≥10% of cells stained, any “plus system”, ≥ 20 fmol/mg, an Allred score of ≥ 3, an immunoreactive score (IRS) ≥ 2, or an H-score ≥ 10, as in a previous EPIC study examining associations with breast cancer subtypes [19]. In the main analyses, we considered individually ER + PR+, ER + PR-, ER-PR- subtypes, and additionally combined the latter two subtypes, because these showed similar association patterns. We did not examine the ER-PR + subtype, because this is rare (only 106 cases) and has questionable reproducibility [20]. In secondary analyses, we divided ER + PR + subtypes and the combined group of ER+/-PR- subtypes according to HER2 status, and examined individually triple negative breast cancer (TNBC, subtype ER-PR-HER2-).

### Statistical analysis

In the main models, we examined ABSI, HI, and BMI on a continuous scale (study-specific z-scores, per one standard deviation increment). To explore non-linearity, we examined in secondary analyses ABSI, HI, and BMI quintile categories (Q1-reference). We estimated hazard ratios (HRs) and 95% confidence intervals (CIs) with delayed-entry Cox proportional hazards models, which are conditional on surviving cancer-free to the start of cancer follow-up and account for left-truncation. The timescale was age in years and women were considered at risk from the date of birth, which defined the origin of the timescale. Cancer follow-up started at the date of the anthropometric assessment, which was considered as baseline in this study, with the following exception. For breast cancers diagnosed at age ≥ 55 years in women younger than 55 years at the anthropometric assessment, cancer follow-up started at age 55 years, as women would have to survive cancer-free to age 55 years to qualify for diagnosis at age ≥ 55 years. Women with follow-up ending before age 55 years were excluded from this analysis (n = 37,158). Cancer follow-up ended at the earliest of the date of diagnosis of the first incident breast cancer (censoring cancers outside the breast and breast cancers with rare morphology at the date of diagnosis), or the date of death, or the date of the last complete follow-up of the corresponding centre, or the date at age 55 years (for the analyses of breast cancers diagnosed at age < 55 years). Women at age ≥ 55 years at the anthropometric assessment (n = 85,482) were excluded from the analyses of breast cancer diagnosed at age < 55 years.

All models were stratified by age at the anthropometric assessment (5-year categories), country, and categories of menopausal status, further dividing post-menopausal women according to age at menopause as follows: pre-menopausal, peri-menopausal, post-menopausal (menopause at age < 46 years), post-menopausal (menopause at age 46 to < 52 years), post-menopausal (menopause at age ≥ 52 years), post-menopausal (menopause at unknown age). All models were adjusted for height, smoking status and intensity, alcohol consumption, physical activity, attained education, HRT use, oral contraceptives use, age at the first period, parity with age at the first live birth, breastfeeding with duration, and log-transformed energy intake assessed at cohort recruitment. Values missing for more than 5% of women were defined as separate categories and the rest were imputed with the median category for the corresponding country and menopausal status (see categories and details in Supplementary Table S1). Statistical significance was evaluated with two-sided p-values from Wald tests for the individual terms.

In subgroup analyses, we examined separately pre-menopausal and post-menopausal women, breast cancers diagnosed at age < 55 years and at age ≥ 55 years, and breast cancer subtypes according to hormone receptor status. To test for heterogeneity, we used the data augmentation method of Lunn and McNeil [21], individually for each anthropometric index (p_heterogeneity_). To test for non-linearity, we compared with a likelihood ratio test the fully adjusted main models, including ABSI, HI, and BMI on a linear untransformed scale, with models including restricted cubic splines, individually for each anthropometric index, with knots at the corresponding quintile boundaries (p_non−linearity_).

To compare traditional and allometric anthropometric indices, we first calculated partial Pearson correlation coefficients (r), with adjustment for age at the anthropometric assessment (continuous), country, and categories of menopausal status and age at menopause. We considered as traditional indices waist and hip circumferences and two sets of residuals, derived individually for each of waist and hip circumferences from linear regression either on BMI (WCadjBMI and HCadjBMI), or on weight and height (WCadjWtHt and HCadjWtHt). We then repeated the main analyses, examining individually waist and hip circumferences and BMI, with adjustment for height and covariates, and combining WCadjWtHt, HCadjWtHt, and BMI, with adjustment for height, and covariates.

To examine the influence of covariates, we omitted all covariates and retained only the stratification and the adjustment for height. To examine possible reverse causality, we excluded women with less than two years of follow-up. To examine the influence of hormone receptor status availability, we repeated the main analyses censoring breast cancers with unknown ERPR status at the date of diagnosis.

We used R version 4.1.3 [22], for data management and generation of figures and tables and Stata-13 [23], for the statistical analyses.

## Results

### Cohort characteristics

During a mean follow up of 14.0 years, 9011 incident breast cancers were diagnosed among 218,276 women. Women in the higher ABSI quintiles were older, more likely to be physically inactive, with no alcohol intake, with primary or no education, post-menopausal, with at least one child, breastfeeding for longer, and never using oral contraceptives compared to women in the lower ABSI quintiles (Table 1). The pattern was similar across waist circumference quintiles, but with a considerably larger difference of 10.3 kg/m^2^ between the mean BMI of Q5 vs. Q1, compared to 1.8 kg/m^2^ for ABSI quintiles (Supplementary Table S1). There were no major differences between women with different breast cancer subtypes, but breast cancers with available ERPR status were diagnosed more recently, in younger women, which had larger ABSI and HI compared to cases with unknown ERPR status (Supplementary Table S2).

**Table 1:** Anthropometric characteristics and breast cancer cases according to ABSI quintiles

Characteristics	ABSI-Q1	ABSI-Q2	ABSI-Q3	ABSI-Q4	ABSI-Q5
Cohort size: n	43,655	43,655	43,656	43,655	43,655
Cases overall: n	1736	1879	1761	1835	1800
ER + PR + subtypes ^a^	498 (64.8)	593 (63.4)	633 (65.5)	710 (69.8)	667 (66.3) ^
ER + PR- subtypes ^a^	114 (14.8)	163 (17.4)	154 (15.9)	145 (14.3)	150 (14.9)
ER-PR- subtypes ^a^	135 (17.6)	163 (17.4)	157 (16.3)	141 (13.9)	163 (16.2)
ERPR unknown ^b^	968 (55.8)	944 (50.2)	795 (45.1)	818 (44.6)	794 (44.1) ^#^
HER2 + subtypes ^a^	99 (19.9)	132 (22.6)	118 (19.8)	120 (18.3)	131 (21.1) ^
HER2- subtypes ^a^	398 (80.1)	452 (77.4)	477 (80.2)	534 (81.7)	491 (78.9)
HER2 unknown ^b^	1239 (71.4)	1295 (68.9)	1166 (66.2)	1181 (64.4)	1178 (65.4) ^#^
Follow-up: years ^c^	13.4 (4.6)	12.9 (4.6)	12.7 (4.6)	12.7 (4.6)	12.7 (4.5) ^#^
Age at diagnosis: years ^c^	60.3 (9.5)	61.5 (9.0)	62.0 (8.9)	62.8 (9.1)	64.3 (8.6) ^#^
Age at baseline: years ^c^	47.2 (11.3)	50.2 (10.3)	51.9 (9.8)	53.3 (9.4)	55.1 (9.4) ^#^
**Anthropometry**					
Height: cm ^c^	162.8 (6.4)	162.3 (6.5)	162.0 (6.6)	161.6 (6.8)	161.1 (7.1) ^#^
Weight: kg ^c^	65.5 (11.0)	64.6 (10.8)	65.5 (11.5)	66.9 (12.1)	68.9 (12.7) ^#^
BMI: kg/m^2 c^	24.8 (4.1)	24.5 (4.1)	25.0 (4.4)	25.6 (4.6)	26.6 (4.8) ^#^
WC: cm ^c^	71.5 (7.4)	75.3 (8.0)	78.8 (8.8)	82.9 (9.6)	90.3 (11.1) ^#^
HC: cm ^c^	98.6 (8.4)	99.3 (8.4)	100.3 (8.8)	101.6 (9.1)	103.6 (9.8) ^#^
ABSI ^c^	66.2 (2.4)	70.3 (0.8)	72.7 (0.7)	75.3 (0.8)	80.2 (3.3) ^#^
HI ^c^	63.9 (3.0)	64.7 (2.5)	65.0 (2.4)	65.1 (2.4)	65.3 (3.0) ^#^
**Lifestyle**					
Never smoker ^d^	24,479 (56.1)	23,674 (54.2)	23,792 (54.5)	24,000 (55.0)	24,227 (55.5) ^#^
Alcohol none ^d^	4246 (9.7)	5589 (12.8)	6737 (15.4)	7682 (17.6)	9488 (21.7) ^#^
Physically inactive ^d^	7325 (16.8)	8435 (19.3)	9551 (21.9)	11,368 (26.0)	14,003 (32.1) ^#^
Education primary ^d^	7672 (17.6)	11,145 (25.5)	13,231 (30.3)	15,983 (36.6)	19,255 (44.1) ^#^
**Reproductive**					
Age at first period ^c^	12.9 (1.5)	13.0 (1.6)	13.1 (1.6)	13.1 (1.6)	13.2 (1.6) ^#^
Age at menopause ^c^	48.5 (5.2)	48.7 (4.9)	48.7 (4.9)	48.7 (4.9)	48.5 (5.1) ^
Post-menopausal ^d^	15,956 (36.6)	19,403 (44.4)	21,986 (50.4)	24,533 (56.2)	28,176 (64.5) ^#^
Nulliparous ^d^	11,069 (25.4)	7697 (17.6)	6448 (14.8)	5657 (13.0)	5676 (13.0) ^#^
Breastfeeding ≥ 6 mo ^d^	13,397 (30.7)	14,649 (33.6)	15,236 (34.9)	15,687 (35.9)	16,475 (37.7) ^#^
OC never ^d^	12,818 (29.4)	15,056 (34.5)	16,833 (38.6)	18,843 (43.2)	21,770 (49.9) ^#^
HRT never ^d^	30,300 (69.4)	28,769 (65.9)	28,866 (66.1)	29,247 (67.0)	30,280 (69.4) ^#^

**ABSI** – a body shape index; **BMI** – body mass index; **ER+/-** – oestrogen receptor status; **HC** – hip circumference; **HER2+/-** – human epidermal receptor 2 status; **HI** – hip index; **n**– number of women or cases; **PR+/-** – progesterone receptor status; **Q1-Q5** – study-specific quintile categories for ABSI (cut-offs: 68.843, 71.525, 73.919, 76.884); **WC** – waist circumference; ^**a**^ – number (percent form cases with available ERPR status (for ERPR) or with available ERPRHER2 status (for HER2) per ABSI quintile); ^b^ – number (percent form total cases per ABSI quintile); ^c^ – mean (standard deviation); ^d^ – number (percent form total women per ABSI quintile); **p-values**: ^ ≥0.05; * <0.05; ^#^ <0.0001 from ANOVA test for continuous variables and χ^2^ test for categorical variables, comparing quintile categories and, for each categorical variable, including all categories shown in Supplementary Table S1

### Associations of allometric anthropometric indices with breast cancer risk

There was no strong evidence for association of ABSI with breast cancer overall (HR = 0.984; 95%CI: 0.961–1.007 per one SD increment; p = 0.162) but there were suggestive weak inverse associations with ABSI in post-menopausal women (HR = 0.971; 95%CI: 0.942-1.000; p = 0.051) and for breast cancers diagnosed at age ≥ 55 years (HR = 0.976; 95%CI: 0.951–1.002; p = 0.067), although with no nominal significance for heterogeneity (Table 2). There were further clear inverse associations of ABSI with both ER + PR- subtypes (HR = 0.894; 95%CI: 0.822–0.971; p = 0.008) and ER-PR- subtypes (HR = 0.906; 95%CI: 0.835–0.983; p = 0.018), but no strong evidence for association with ER + PR + subtypes (HR = 0.971; 95%CI: 0.933–1.010; p = 0.141), with p_heterogeneity_=0.037 for the comparison of ER+/-PR- combined vs. ER + PR + subtypes. There was no evidence for heterogeneity according to HER2 status for ER + PR + subtypes (p_heterogeneity_=0.390) or for ER+/-PR- subtypes (p_heterogeneity_=0.680). HI was not associated with breast cancer overall (HR = 1.013; 95%CI: 0.990–1.036 per one SD; p = 0.276) or within subgroups by menopausal status at the anthropometric assessment or by age at diagnosis, or with any individual hormone receptor subtypes. BMI was associated strongly positively with breast cancer overall (HR = 1.074; 95%CI: 1.049–1.098 per one SD; p < 0.001), specifically for post-menopausal women (HR = 1.117; 95%CI: 1.085–1.150, p < 0.001, p_heterogeneity_<0.001), for cancers diagnosed at age ≥ 55 years (HR = 1.104; 95%CI: 1.076–1.132, p < 0.001, p_heterogeneity_<0.001), and for ER + PR + subtypes (HR = 1.122; 95%CI: 1.080–1.165; p < 0.001), but not for ER + PR- or ER-PR- subtypes (p_heterogeneity_<0.001 for ER+/-PR- vs. ER + PR+), with no evidence for heterogeneity according to HER2 status for ER + PR + subtypes (p_heterogeneity_=0.449) or for ER+/-PR- subtypes (p_heterogeneity_=0.693) (Table 2).

**Table 2:** Associations of continuous allometric anthropometric indices with breast cancer risk

		ABSI (per one SD increase)		HI (per one SD increase)		BMI (per one SD increase)	
	Cases	HR (95% CI)	p	HR (95% CI)	p	HR (95% CI)	p
Overall	9011	0.984 (0.961–1.007)	0.162	1.013 (0.990–1.036)	0.276	1.074 (1.049–1.098)	< 0.001
**MP baseline**							
Pre-MP	2178	0.988 (0.941–1.038)	0.643	1.028 (0.982–1.075)	0.239	0.980 (0.932–1.030)	0.422
Post-MP	5268	0.971 (0.942-1.000)	0.051	1.011 (0.981–1.041)	0.490	1.117 (1.085–1.150)	< 0.001
p _heterogeneity_		0.535		0.543		< 0.001	
**Age diagnosis**							
<55 years	1968	1.006 (0.956–1.059)	0.817	1.026 (0.979–1.076)	0.285	0.966 (0.917–1.019)	0.206
≥55 years	7043	0.976 (0.951–1.002)	0.067	1.006 (0.981–1.032)	0.630	1.104 (1.076–1.132)	< 0.001
p _heterogeneity_		0.299		0.476		< 0.001	
**ERPR status**							
ER + PR+	3101	0.971 (0.933–1.010)	0.141	1.016 (0.978–1.056)	0.409	1.122 (1.080–1.165)	< 0.001
ER + PR-	726	0.894 (0.822–0.971)	0.008	0.981 (0.905–1.063)	0.644	0.998 (0.917–1.086)	0.956
ER-PR-	759	0.906 (0.835–0.983)	0.018	1.028 (0.951–1.112)	0.487	0.996 (0.919–1.081)	0.933
p _heterogeneity_		0.108		0.682		0.004	
ER+/-PR-	1485	0.900 (0.849–0.954)	< 0.001	1.006 (0.951–1.064)	0.842	0.997 (0.940–1.057)	0.924
p _ER+/−PR− vs. ER+PR+_		0.037		0.763		< 0.001	
**HER2 status**							
ER + PR + HER2+	288	1.042 (0.918–1.183)	0.526	0.972 (0.860–1.098)	0.647	1.206 (1.073–1.356)	0.002
ER + PR + HER2-	1690	0.981 (0.929–1.035)	0.474	1.016 (0.965–1.071)	0.542	1.148 (1.091–1.207)	< 0.001
p _heterogeneity_		0.390		0.511		0.449	
ER+/-PR-HER2+	298	0.934 (0.822–1.062)	0.297	0.970 (0.858–1.096)	0.624	1.045 (0.921–1.185)	0.493
ER+/-PR-HER2-	626	0.904 (0.826–0.989)	0.027	1.017 (0.932–1.109)	0.703	1.013 (0.926–1.108)	0.778
p _heterogeneity_		0.680		0.536		0.693	
TNBC	314	0.927 (0.817–1.051)	0.238	1.046 (0.925–1.182)	0.473	0.993 (0.874–1.127)	0.908

**ABSI** – a body shape index; **BMI** – body mass index; **CI** – confidence interval; **ER+/-** – oestrogen receptor status; **HER2+/-** – human epidermal receptor 2 status; **HI** – hip index; **HR** – hazard ratio; **p-value** – from Wald test for the individual term; **Pre-MP** – pre-menopausal at baseline (the day of the anthropometric assessment); **Post-MP** – post-menopausal at baseline; **PR+/-** – progesterone receptor status; **SD** – standard deviation; **TNBC** – triple-negative breast cancer (ER-PR-HER2- subtype); **HR (95% CI)** (per one SD increase) were derived from Cox proportional hazards models, including ABSI, HI, and BMI on a continuous scale (z-scores, value minus mean (72.939 for ABSI; 64.806 for HI; 25.304 for BMI), divided by SD (5.070 for ABSI; 2.717 for HI; 4.464 for BMI)) as exposures, stratified by age at the anthropometric assessment, country, and categories of menopausal status and, for post-menopausal women, age at menopause (pre-menopausal, peri-menopausal, menopause at < 46 years, menopause at 46 to < 52 years, menopause at ≥ 52 years, menopause at unknown age), and adjusted for height (continuous), smoking status and intensity (never smoker, former quit ≥ 15 years, former quit < 15 years, current ≤ 10 cigarettes/day, current > 10 cigarettes/day), alcohol consumption (none, < 4 g/day, 4 to < 16 g/day, ≥ 16 g/day), physical activity (inactive, moderately inactive, moderately active, active), education (primary/none, technical, secondary, university/longer), hormone replacement therapy use (never, former, current, missing), oral contraceptives use (never, former, current), age at the first period (continuous), parity with age at first live birth (nulliparous, one at < 25 years, one at ≥ 25 years, two at < 25 years, two at ≥ 25 years, ≥ 3 at < 25 years, ≥ 3 at ≥ 25 years, missing), breastfeeding with duration (never, < 6 months, ≥ 6 months, missing), energy intake (log-transformed continuous); **p**_**heterogeneity**_ and **p**_**ER+/−PR− vs. ER+PR+**_ were derived with the data augmentation method of Lunn and McNeil [21]

**Fig. 1:**
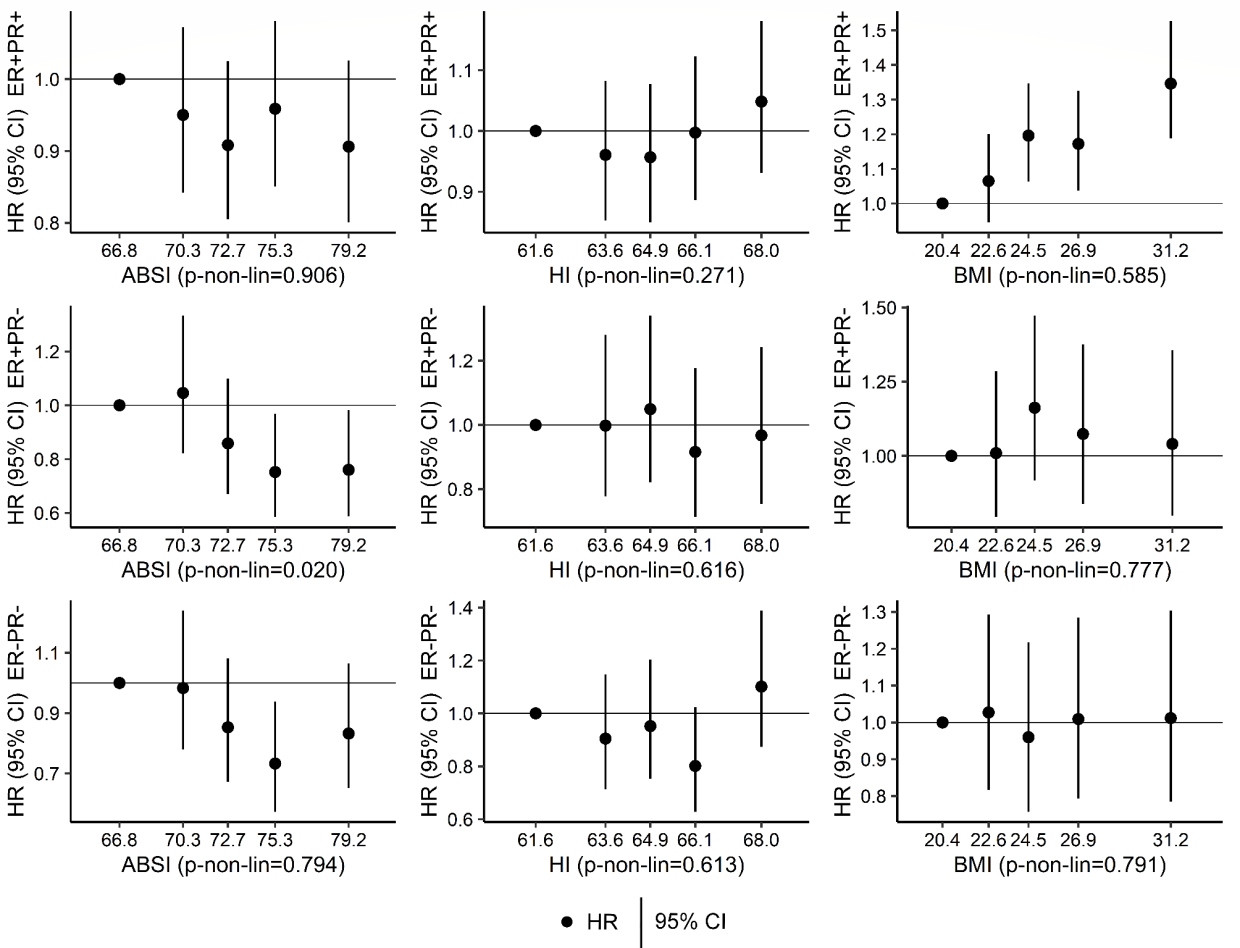
Breast cancer risk according to allometric anthropometric index quintiles **ABSI – a body shape index; BMI – body mass index; CI – confidence interval; ER+/- – oestrogen receptor status; HI – hip index; HR – hazard ratio; PR+/- – progesterone receptor status. HR (95% CI)** (compared to the lowest quintile) were derived from Cox proportional hazards models, including quintile categories for ASBI, HI, and BMI as exposures (lowest quintile reference, cut-offs: 68.843, 71.525, 73.919, 76.884 for ABSI; 62.763, 64.240, 65.477, 66.933 for HI; 21.63, 23.54, 25.58, 28.55 kg/m^2^ for BMI). Estimates are plotted at the quintile medians. Models were stratified by age at the anthropometric assessment, country, and categories of menopausal status and, for post-menopausal women, age at menopause (pre-menopausal, peri-menopausal, menopause at < 46 years, menopause at 46 to < 52 years, menopause at ≥ 52 years, menopause at unknown age), and adjusted for height (continuous), smoking status and intensity (never smoker, former quit ≥ 15 years, former quit < 15 years, current ≤ 10 cigarettes/day, current > 10 cigarettes/day), alcohol consumption (none, < 4 g/day, 4 to < 16 g/day, ≥ 16 g/day), physical activity (inactive, moderately inactive, moderately active, active), education (primary/none, technical, secondary, university/longer), hormone replacement therapy use (never, former, current, missing), oral contraceptives use (never, former, current), age at the first period (continuous), parity with age at the first live birth (nulliparous, one at < 25 years, one at ≥ 25 years, two at < 25 years, two at ≥ 25 years, ≥ 3 at < 25 years, ≥ 3 at ≥ 25 years, missing), breastfeeding with duration (never, < 6 months, ≥ 6 months, missing), and energy intake (log-transformed continuous); **p-non-lin.** – p-value for non-linearity, obtained from likelihood ratio tests comparing the fully adjusted models, including ABSI, HI, and BMI on a linear untransformed scale, with models including restricted cubic splines, individually for each anthropometric index, with knots at the corresponding quintile cut-offs. HR estimates are shown in Supplementary Table S3

Examining quintile categories, there was some evidence for non-linearity of the inverse associations of ABSI with ER + PR- subtypes (p_non−linearity_=0.020), with the lowest HRs for Q4 vs. Q1 for both ER + PR- and ER-PR- subtypes, but with no evidence for association of Q2 vs. Q1 (Fig. 1, Supplementary Table S3 for full list of model estimates). There was little evidence, however, for non-linearity of any of the positive associations with BMI (Fig. 1, Supplementary Table S3).

### Comparisons of traditional and allometric anthropometric indices

Waist and hip circumferences were correlated strongly positively with BMI (r_WC_=0.84; r_HC_=0.85) but only weakly positively with height (r_WC_=0.06; r_HC_=0.14) (Supplementary Table S4). The residuals of waist and hip circumferences adjusted for BMI were correlated weakly with BMI, but more strongly positively with height (r_WCadjBMI_=0.32; r_HCadjBMI_=0.47). As the stronger positive correlations with height of residuals adjusted only for BMI resulted from the transformation and were thus artificial, we did not examine further their associations with breast cancer risk. The residuals of waist and hip circumferences adjusted for weight and height were corelated weakly not only with BMI but also with height (r_WCadjWtHt_=0.12; r_HCadjWtHt_=0.09) and were each correlated very strongly positively with the corresponding allometric index, ABSI or HI (r ≥ 0.97). ABSI and HI were correlated minimally with BMI and height (r within +/-0.06) (Supplementary Table S4).

**Fig. 2:**
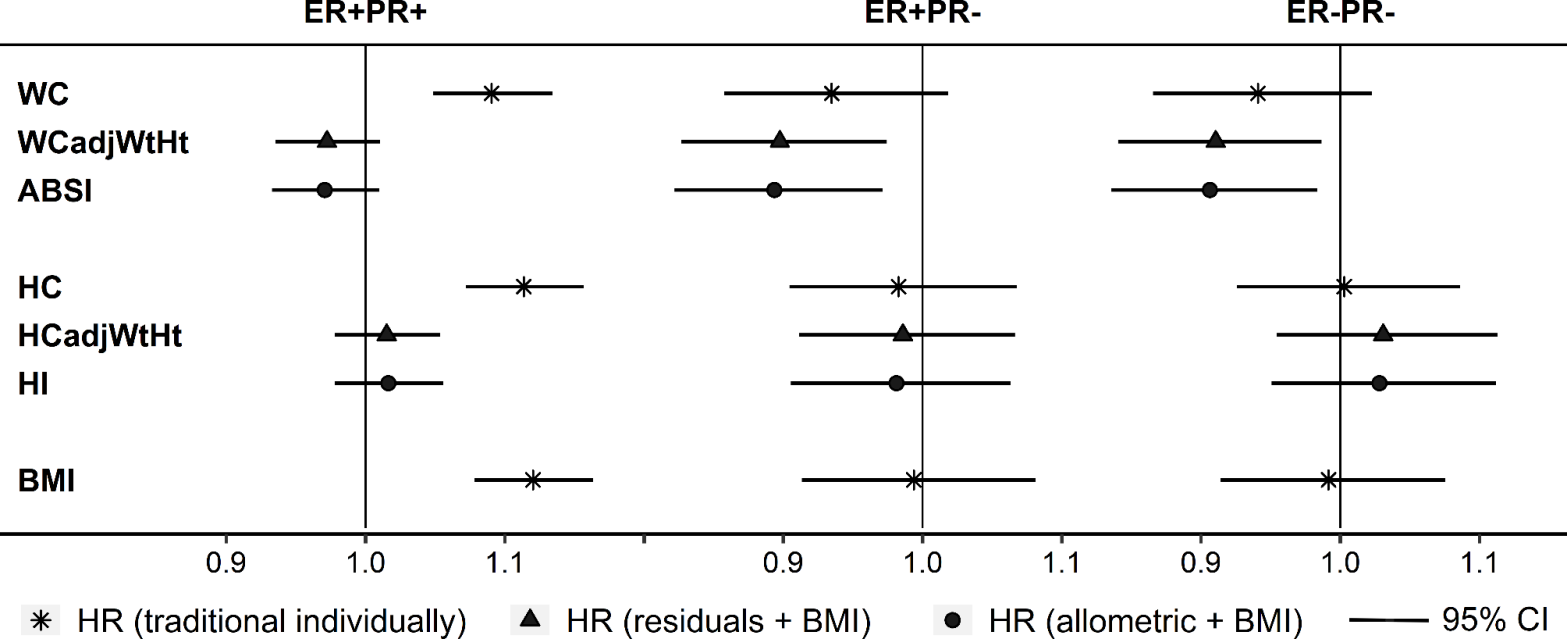
Comparisons of traditional and allometric anthropometric indices with respect to breast cancer risk **ABSI** – a body shape index; **BMI** – body mass index; **CI** – confidence interval; **ER+/-** – oestrogen receptor status; **HC** – hip circumference; **HCadjWtHt** – residuals of hip circumference from linear regression on weight and height; **HI** – hip index; **HR** – hazard ratio; **PR+/-** – progesterone receptor status; **SD** – standard deviation; **WC** – waist circumference; **WCadjWtHt** – residuals of waist circumference from linear regression on weight and height Models include the following exposures on a continuous scale (z-scores, value minus mean divided by SD): ABSI (mean 72.939; SD 5.070), HI (mean 64.806; SD 2.717), and BMI (mean 25.304; SD 4.464) **(allometric + BMI)**; WCadjWtHt (mean 0; SD 5.641), HCadjWtHt (mean 0; SD 4.246), and BMI **(residuals + BMI)**; one of WC (mean 79.762; SD 11.162), or HC (mean 100.694; SD 9.096), or BMI, each in a separate model **(traditional individually)**; **HR (95% CI)** (per one SD increase) were derived from Cox proportional hazards models, stratified by age at the anthropometric assessment, country, and categories of menopausal status and, for post-menopausal women, age at menopause (pre-menopausal, peri-menopausal, menopause at < 46 years, menopause at 46 to < 52 years, menopause at ≥ 52 years, menopause at unknown age), and adjusted for height (continuous), smoking status and intensity (never smoker, former quit ≥ 15 years, former quit < 15 years, current ≤ 10 cigarettes/day, current > 10 cigarettes/day), alcohol consumption (none, < 4 g/day, 4 to < 16 g/day, ≥ 16 g/day), physical activity (inactive, moderately inactive, moderately active, active), education (primary/none, technical, secondary, university/longer), hormone replacement therapy use (never, former, current, missing), oral contraceptives use (never, former, current), age at the first period (continuous), parity with age at the first live birth (nulliparous, one at < 25 years, one at ≥ 25 years, two at < 25 years, two at ≥ 25 years, ≥ 3 at < 25 years, ≥ 3 at ≥ 25 years, missing), breastfeeding with duration (never, < 6 months, ≥ 6 months, missing), and energy intake (log-transformed continuous)

Corresponding to their strong positive correlations with BMI, both waist and hip circumferences, similarly to BMI, were associated strongly positively with ER + PR + subtypes, although the positive association with waist circumference was slightly attenuated compared to the corresponding association with BMI (Fig. 2). The association pattern was similar for breast cancer overall, for post-menopausal women, and for cancers diagnosed at age ≥ 55 years (Supplementary Table S5). As BMI was not associated with ER + PR- or ER-PR- subtypes, hip circumference was not associated with them either, while waist circumference showed associations in the inverse direction but attenuated towards the null compared to the corresponding associations with ABSI (Fig. 2), and with nominal statistical significance only for ER+/-PR- combined (Supplementary Table S5). Association estimates based on residuals of waist and hip circumferences adjusted for weight and height showed no material difference to the corresponding estimates based on ABSI and HI (Fig. 2).

### Sensitivity analyses

Omitting the adjustment for covariates from the main models or restricting the analyses to women with follow-up ≥ 2 years influenced little the inverse associations with ABSI, but restricting the analyses to cancers with known ERPR status resulted in stronger inverse associations with ABSI, even for breast cancer overall (HR = 0.948; 95%CI: 0.918–0.980; p = 0.001) (Supplementary Table S6). Omitting the adjustment for covariates slightly attenuated the positive associations with BMI, while restricting the analyses to women with follow-up ≥ 2 years or to cancers with known ERPR status had little influence on the positive associations with BMI. For breast cancers diagnosed at age < 55 years, however, an inverse association with BMI was noted in the minimally adjusted model (HR = 0.942; 95%CI: 0.895–0.991; p = 0.021) and for cancers with known ERPR status (HR = 0.910; 95%CI: 0.844–0.981; p = 0.014) (Supplementary Table S6).

## Discussion

In our study, ABSI was associated inversely with post-menopausal PR- breast cancer subtypes, irrespective of ER and HER2 status, while HI showed no material associations. BMI was associated strongly positively with ER + PR + but not with PR- subtypes. Waist and hip circumferences resembled the associations of BMI, while residuals of waist and hip circumferences adjusted for weight and height were identical to the corresponding associations of ABSI and HI with breast cancer risk.

To our knowledge, this is the first large prospective study to report an inverse association of ABSI with post-menopausal PR- breast cancer subtypes, which clearly differs from the positive associations of ABSI with other obesity-related cancers [4]. ABSI was not associated with breast cancer overall, in agreement with previous studies examining ABSI [4, 24, 25], or waist circumference and the waist-to-hip ratio [1], or residuals of waist circumference adjusted for BMI [26]. Our findings, however, differ from studies reporting positive associations with waist circumference or the waist-to-hip ratio [27, 28], because the associations with traditional waist and hip size measures are driven by their strong correlation with BMI [4]. Our findings also differ from the positive association of ABSI with breast cancer overall reported by Parra-Soto et al. [10] for UK Biobank, possibly due to differences in the definition of incident cancers and the influence of breast cancers diagnosed subsequently to cancers in other locations (which in our studies, the current and [4], were censored at the date of diagnosis of the first incident cancer outside the breast), or due to the inclusion of other ethnicities in their study. Although an inverse association of ABSI has been reported for post-menopausal breast cancer, specifically in HRT users [11], this population-based case-control study recruited cases within one year after cancer diagnosis and treatment, which may have influenced the results due to reverse causation. To our knowledge, no studies have previously examined associations of HI with breast cancer risk, except our UK Biobank study [4], which similarly to this study, found no evidence for association with breast cancer risk. Therefore, our results do not support previous reports of positive associations of hip circumference with pre-menopausal breast cancer [29, 30].

The inverse association of ABSI with post-menopausal breast cancer risk is unusual and unexpected. This is, however, plausible because large abdominal size is a hall-mark not only of metabolic dysfunction [8], but also of chronic glucocorticoid excess [31], and metabolic factors and glucocorticoids appear to show opposite effects on breast cancer development. Although hyperinsulinaemia and insulin resistance in post-menopausal women are associated with higher risk of ER + breast cancer subtypes [9, 32], use of exogenous glucocorticoids has been associated with lower risk of ER + PR + subtypes [33]. At cellular level, chronic insulin exposure facilitates oestradiol-dependent growth of ER + breast cancer cell lines [34], while glucocorticoids suppress this insulin induction [35]. Glucocorticoids also inhibit cell proliferation and cancer cell growth induced by oestradiol and progesterone [36–38] and glucocorticoid receptor (GR) positivity of ER + PR+ and ER + PR - subtypes is associated with longer relapse-free survival [39]. Glucocorticoids additionally reduce oestradiol availability via GR-mediated activation of oestrogen sulfotransferase (SULT1E1) [40] and circulating oestradiol is associated inversely with ABSI [41]. Nevertheless, GR-positivity is lower for ER- and PR- breast cancer subtypes [42], glucocorticoids promote tumour growth and invasion in TNBC cell lines [43–45], and higher GR gene expression in TNBC is associated with a shorter relapse-free period [45, 46]. In our study, however, we found little evidence for heterogeneity of the inverse association with ABSI according to HER2 status and no evidence for a positive association of ABSI with TNBC, although information for HER2 status was limited. An alternative mechanism explaining the inverse association of ABSI with ER-PR-, as well as with ER + PR- subtypes, could involve suppression of the oncogenic phosphoinositide 3-kinase (PI3K)/protein kinase B (Akt)/mammalian target of rapamycin (mTOR) pathway (PI3K/Akt/mTOR), which is prominent in both subtypes [47]. The PI3K/Akt/mTOR pathway is involved in adipocyte-mediated proliferation and migration of breast cancer cells [48] and can be disrupted by glucocorticoids [49, 50].

It is likely, therefore, that the net outcome for breast cancer development depends on the balance between the opposing actions of insulin, oestradiol, progesterone, glucocorticoids, and other alternative pathways. A different balance of ABSI-related factors within different populations and datasets may explain why the inverse association with ABSI was more prominent for the subset of cancers with available ERPR status in our study and why other studies have either failed to find evidence for association, or have reported a positive association with ABSI.

A major strength of our study is the availability of waist and hip size measurements from a large multicenter cohort. Our study included women with a variety of lifestyle, reproductive, and dietary patterns from eight European countries and a sizeable number of incident breast cancer cases. A major limitation of our study is the unavailability of hormone receptor status for part of the cases, resulting in a relatively smaller sample size for PR- subtypes and an even smaller sample size for HER2 status, because the routine determination of HER2 status is more recent and was missing for a large proportion of women. A further limitation is the lack of information for menopausal status at the time of cancer diagnosis, which confined us to classifying cancers by age at diagnosis, although women post-menopausal at the anthropometric assessment were clearly post-menopausal at cancer diagnosis. Menopausal status in women from France may also have changed from cohort recruitment to the anthropometric assessment, some three years later, potentially introducing misclassification bias, but we did correct menopausal status for women from France according to age at the anthropometric assessment. In addition, similarly to all prospective studies using exposures assessed at a single time-point, we were limited to assuming that body shape and body size assessed near cohort recruitment had remained the same throughout the follow-up period.

## Conclusions

In the EPIC cohort, ABSI was not associated with breast cancer risk overall, but was associated inversely with post-menopausal breast cancer and PR- subtypes. Our findings require validation in other cohorts and with a larger number of PR- breast cancer cases but suggest that a competition between ABSI-related factors such as glucocorticoids, sex steroids, and insulin resistance determines the net outcome for the risk of individual breast cancer subtypes.

## Electronic supplementary material

Below is the link to the electronic supplementary material.

Supplementary Material 1

## Data Availability

The dataset analysed in the current study was used under license and cannot be made freely available in a public repository or obtained from the authors due to restrictions related to privacy regulations and informed consent of the participants. Access to the data, however, can be obtained by *bona fide* researchers from the central EPIC repository at IARC, subject to approval of the research project by the EPIC Steering Committee and a material transfer agreement. For information on how to submit an application for gaining access to EPIC data and/or biospecimens from the EPIC repository at IARC, please follow the instructions at https://epic.iarc.fr/access/index.php For further queries related to the data used in this study, contact the corresponding author Dr Sofia Christakoudi s.christakoudi@imperial.ac.uk.

## Abbreviations

ABSI: a body shape index
BMI: body mass index
CI: confidence interval
EPIC: European Prospective Investigation into Cancer and Nutrition
ER: oestrogen receptor
GR: glucocorticoid receptor
HER2: human epidermal receptor 2
HI: hip index
HR: hazard ratio
HRT: hormone replacement therapy
ICD-O: International Classification of Diseases for Oncology
IRS: immunoreactive score
PI3K/Akt/mTOR: phosphoinositide 3-kinase (PI3K)/ protein kinase B (Akt)/ mammalian target of rapamycin (mTOR)
PR: progesterone receptor
SD: standard deviation
SULT1E1: oestrogen sulfotransferase
TNBC: triple-negative breast cancer
UK: United Kingdom
