# Supplementary material for: A body shape index (ABSI) is associated inversely with post-menopausal progesterone-receptor-negative breast cancer risk in a large European cohort
Source: BMC Cancer. 2023 Jun 19;23:562. doi: 10.1186/s12885-023-11056-1 (PMC10278318; doi:10.1186/s12885-023-11056-1)
Supplement: Supplementary file 1 — Supplementary Material 1 [file 12885_2023_11056_MOESM1_ESM.pdf]

# A body shape index (ABSI) is associated inversely with progesterone receptor negative breast cancer risk in a large European cohort

Christakoudi S, Tsilidis KK, Dossus L, Rinaldi S, Weiderpass E, Antoniussen CS, Dahm CC, Tjønneland A, Mellemkjær L, Katzke V, Kaaks R, Schulze MB, Masala G, Grioni S, Panico S, Tumino R, Sacerdote C, May AM, Monninkhof EM, Quirós JR, Bonet C, Sánchez MJ, Amiano P, Chirlaque MD, Guevara M, Rosendahl AH, Stocks T, Perez-Cornago A, Tin Tin S, Heath AK, Aglago EK, Peruchet-Noray L, Freisling H, Riboli E

**Supplementary Figures**

Supplementary Figure S1 Flow diagram of EPIC participants included in the study .................. 2

**Supplementary Tables**

Supplementary Table S1 Characteristics of women according to quintiles of ABSI and waist circumference ........................................................................ 3

Supplementary Table S2 Characteristics of breast cancer cases according to hormone receptor status........................................................................................ 7

Supplementary Table S3 Breast cancer risk according to allometric anthropometric index quintiles ........................................................................................ 10

Supplementary Table S4 Correlations between traditional and allometric anthropometric indices ........................................................................................ 13

Supplementary Table S5 Comparisons of traditional and allometric anthropometric indices with respect to breast cancer risk......................................................... 14

Supplementary Table S6 Associations of allometric anthropometric indices with breast cancer risk (sensitivity analyses)................................................................ 16

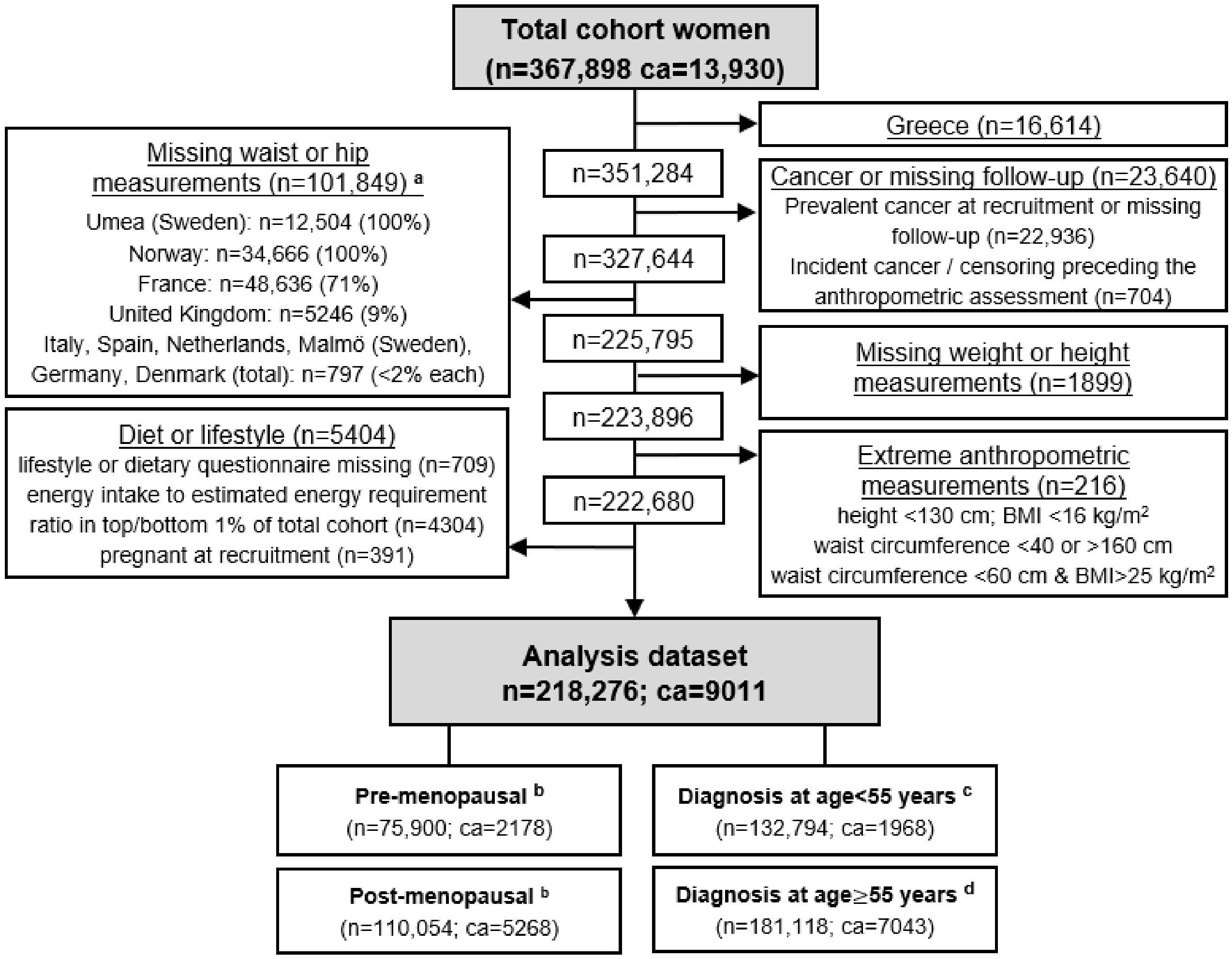


**Supplementary Figure S1 Flow diagram of EPIC participants included in the study**

**BMI** – body mass index; **ca** – breast cancer cases; **EPIC** – European Prospective Investigation into Cancer and Nutrition cohort; **n** – number of women;

[a] – missing anthropometric measurements within one year of cohort recruitment, except for France, where all measurements were collected on average 3.8 years later (standard deviation 1.3 years) (percentage from total available at this step per country, or for Sweden per centre);

[b] – based on a centrally derived variable included in the EPIC dataset [3], for which women with menstruation at cohort recruitment were considered pre-menopausal with at least ten periods per year and without current hormone replacement therapy or oral contraceptives use, or peri-menopausal otherwise, and women without menstruation (including due to bilateral oophorectomy) and without hysterectomy were considered post-menopausal. Age cut-offs were used for women with hysterectomy, for women using hormones, and when information was missing (<46 years at recruitment for pre-menopausal, 46 to <55 years at recruitment for peri-menopausal, ≥55 years at recruitment for post-menopausal);

[c] – women with anthropometric assessment at age ≥55 years were excluded;

[d] – women with end of cancer follow-up at age<55 years were excluded.

**Supplementary Table S1 Characteristics of women according to quintiles of ABSI and waist circumference**

| Characteristics | ABSI | | | | | Waist circumference | | | | |
|---|---|---|---|---|---|---|---|---|---|---|
| | Q1 | Q2 | Q3 | Q4 | Q5 | Q1 | Q2 | Q3 | Q4 | Q5 |
| Cohort size: n | 43,655 | 43,655 | 43,656 | 43,655 | 43,655 | 44,667 | 42,745 | 47,110 | 40,178 | 43,576 |
| Age at baseline: years [a] | 47.2 (11.3) | 50.2 (10.3) | 51.9 ( 9.8) | 53.3 ( 9.4) | 55.1 ( 9.4) # | 46.4 (11.6) | 50.2 (10.4) | 52.6 ( 9.7) | 53.9 ( 9.4) | 54.7 ( 8.6) # |
| **Cases: n (%)** | | | | | | | | | | |
| Overall | 1736 | 1879 | 1761 | 1835 | 1800 | 1593 | 1737 | 2067 | 1706 | 1908 |
| ER+PR+ subtypes [b] | 498 (64.8) | 593 (63.4) | 633 (65.5) | 710 (69.8) | 667 (66.3) ^ | 494 (62.4) | 577 (65.5) | 700 (64.9) | 614 (68.7) | 716 (68.4) * |
| ER+PR- subtypes [b] | 114 (14.8) | 163 (17.4) | 154 (15.9) | 145 (14.3) | 150 (14.9) | 133 (16.8) | 124 (14.1) | 187 (17.3) | 135 (15.1) | 147 (14.0) |
| ER-PR- subtypes [b] | 135 (17.6) | 163 (17.4) | 157 (16.3) | 141 (13.9) | 163 (16.2) | 138 (17.4) | 156 (17.7) | 168 (15.6) | 128 (14.3) | 169 (16.1) |
| ERPR unknown [c] | 968 (55.8) | 944 (50.2) | 795 (45.1) | 818 (44.6) | 794 (44.1) # | 801 (50.3) | 856 (49.3) | 989 (47.8) | 812 (47.6) | 861 (45.1) * |
| HER2+ subtypes [b] | 99 (19.9) | 132 (22.6) | 118 (19.8) | 120 (18.3) | 131 (21.1) ^ | 100 (21) | 111 (20.9) | 135 (20.1) | 102 (17.6) | 152 (21.8) ^ |
| HER2- subtypes [b] | 398 (80.1) | 452 (77.4) | 477 (80.2) | 534 (81.7) | 491 (78.9) | 376 (79) | 421 (79.1) | 535 (79.9) | 476 (82.4) | 544 (78.2) |
| HER2 unknown [c] | 1239 (71.4) | 1295 (68.9) | 1166 (66.2) | 1181 (64.4) | 1178 (65.4) # | 1117 (70.1) | 1205 (69.4) | 1397 (67.6) | 1128 (66.1) | 1212 (63.5)* |
| Follow-up: years [a] | 13.4 (4.6) | 12.9 (4.6) | 12.7 (4.6) | 12.7 (4.6) | 12.7 (4.5) # | 12.9 (4.6) | 12.8 (4.6) | 12.8 (4.6) | 12.9 (4.5) | 12.9 (4.6) ^ |
| Age at diagnosis: years [a] | 60.3 (9.5) | 61.5 (9.0) | 62.0 (8.9) | 62.8 (9.1) | 64.3 (8.6) # | 58.8 (9.5) | 60.7 (9.3) | 62.8 (8.8) | 63.8 (8.7) | 64.3 (8.3) # |
| **Anthropometry: mean (SD)** | | | | | | | | | | |
| Height: cm | 162.8 (6.4) | 162.3 (6.5) | 162.0 (6.6) | 161.6 (6.8) | 161.1 (7.1) # | 162.3 (6.3) | 162.6 (6.5) | 162.3 (6.7) | 161.7 (6.8) | 160.9 (7.0) # |
| Weight: kg | 65.5 (11.0) | 64.6 (10.8) | 65.5 (11.5) | 66.9 (12.1) | 68.9 (12.7) # | 55.4 (5.9) | 60.6 ( 6.0) | 64.9 (6.5) | 70.0 (7.2) | 81.1 (11.7) # |
| BMI: kg/m$^2$ | 24.8 (4.1) | 24.5 (4.1) | 25.0 (4.4) | 25.6 (4.6) | 26.6 (4.8) # | 21.0 (1.9) | 22.9 (2.0) | 24.7 (2.2) | 26.8 (2.5) | 31.3 (4.3) # |
| WC: cm | 71.5 ( 7.4) | 75.3 ( 8.0) | 78.8 ( 8.8) | 82.9 ( 9.6) | 90.3 (11.1) # | 66.7 (2.8) | 72.8 (1.4) | 78.2 (1.7) | 84.7 (2.1) | 97.1 (7.6) # |
| HC: cm | 98.6 (8.4) | 99.3 (8.4) | 100.3 (8.8) | 101.6 (9.1) | 103.6 (9.8) # | 92.1 (4.8) | 96.4 (4.7) | 99.8 (5.1) | 103.6 (5.5) | 111.9 (9.2) # |
| ABSI | 66.2 (2.4) | 70.3 (0.8) | 72.7 (0.7) | 75.3 (0.8) | 80.2 (3.3) # | 68.9 (3.8) | 71.1 (3.7) | 72.8 (3.9) | 74.7 (4.2) | 77.5 (5.0) # |
| HI | 63.9 (3.0) | 64.7 (2.5) | 65.0 (2.4) | 65.1 (2.4) | 65.3 (3.0) # | 64.6 (2.8) | 64.8 (2.6) | 64.8 (2.5) | 64.8 (2.5) | 65.1 (3.0) # |
| **Smoking: n (%)** [c] | | | | | | | | | | |
| Never smoker | 24,479 (56.1) | 23,674 (54.2) | 23,792 (54.5) | 24,000 (55.0) | 24,227 (55.5) # | 24,555 (55.0) | 22,719 (53.2) | 25,051 (53.2) | 22,121 (55.1) | 25,726 (59.0) # |
| Former: quit ≥15 years | 5039 (11.5) | 5090 (11.7) | 4965 (11.4) | 4705 (10.8) | 4219 (9.7) | 4691 (10.5) | 4912 (11.5) | 5606 (11.9) | 4551 (11.3) | 4258 (9.8) |
| Former: quit <15 years | 5919 (13.6) | 5515 (12.6) | 5260 (12.0) | 4813 (11.0) | 4703 (10.8) | 5269 (11.8) | 5494 (12.9) | 5733 (12.2) | 4754 (11.8) | 4960 (11.4) |
| Former: time missing [d] | 339 (0.8) | 354 (0.8) | 333 (0.8) | 363 (0.8) | 349 (0.8) | 342 (0.8) | 361 (0.8) | 384 (0.8) | 330 (0.8) | 321 (0.7) |
| Current: ≤10 cig./day | 3582 (8.2) | 3988 (9.1) | 3888 (8.9) | 3886 (8.9) | 3718 (8.5) | 4435 (9.9) | 4007 (9.4) | 4269 (9.1) | 3315 (8.3) | 3036 (7.0) |
| Current: >10 cig./day | 3600 (8.2) | 4272 (9.8) | 4683 (10.7) | 5193 (11.9) | 5833 (13.4) | 4518 (10.1) | 4485 (10.5) | 5320 (11.3) | 4501 (11.2) | 4757 (10.9) |
| Current: cig. missing [d] | 314 (0.7) | 290 (0.7) | 243 (0.6) | 247 (0.6) | 216 (0.5) | 344 (0.8) | 296 (0.7) | 285 (0.6) | 209 (0.5) | 176 (0.4) |
| Missing [d] | 383 (0.9) | 472 (1.1) | 492 (1.1) | 448 (1.0) | 390 (0.9) | 513 (1.1) | 471 (1.1) | 462 (1.0) | 397 (1.0) | 342 (0.8) |

| Characteristics | ABSI | | | | | Waist circumference | | | | |
|---|---|---|---|---|---|---|---|---|---|---|
| | Q1 | Q2 | Q3 | Q4 | Q5 | Q1 | Q2 | Q3 | Q4 | Q5 |
| **Alcohol: n (%)** [c] | | | | | | | | | | |
| None | 4246 (9.7) | 5589 (12.8) | 6737 (15.4) | 7682 (17.6) | 9488 (21.7) [#] | 4050 (9.1) | 4663 (10.9) | 6573 (14.0) | 7377 (18.4) | 11,079 (25.4) [#] |
| Light: <4 g/day | 15,862 (36.3) | 14,719 (33.7) | 14,215 (32.6) | 14,172 (32.5) | 13,445 (30.8) | 15,451 (34.6) | 13,964 (32.7) | 15,221 (32.3) | 13,080 (32.6) | 14,697 (33.7) |
| Moderate: 4 to <16 g/day | 17,288 (39.6) | 16,169 (37.0) | 15,012 (34.4) | 13,690 (31.4) | 12,484 (28.6) | 17,795 (39.8) | 16,148 (37.8) | 16,581 (35.2) | 12,630 (31.4) | 11,489 (26.4) |
| Heavy: ≥16 g/day | 6259 (14.3) | 7178 (16.4) | 7692 (17.6) | 8111 (18.6) | 8238 (18.9) | 7371 (16.5) | 7970 (18.6) | 8735 (18.5) | 7091 (17.6) | 6311 (14.5) |
| **Physical activity: n (%)** [c] | | | | | | | | | | |
| Inactive | 7325 (16.8) | 8435 (19.3) | 9551 (21.9) | 11,368 (26.0) | 14,003 (32.1) [#] | 6688 (15.0) | 7638 (17.9) | 10,325 (21.9) | 10,819 (26.9) | 15,212 (34.9) [#] |
| Moderately inactive | 15,218 (34.9) | 15,570 (35.7) | 15,532 (35.6) | 15,333 (35.1) | 15,058 (34.5) | 15,937 (35.7) | 15,519 (36.3) | 16,810 (35.7) | 14,040 (34.9) | 14,405 (33.1) |
| Moderately active | 11200 (25.7) | 10,380 (23.8) | 10,060 (23.0) | 9197 (21.1) | 7795 (17.9) | 12,001 (26.9) | 10,648 (24.9) | 10,638 (22.6) | 8109 (20.2) | 7236 (16.6) |
| Active | 8979 (20.6) | 8475 (19.4) | 7814 (17.9) | 7162 (16.4) | 6174 (14.1) | 9219 (20.6) | 8244 (19.3) | 8575 (18.2) | 6530 (16.3) | 6036 (13.9) |
| Missing [e] | 933 (2.1) | 795 (1.8) | 699 (1.6) | 595 (1.4) | 625 (1.4) | 822 (1.8) | 696 (1.6) | 762 (1.6) | 680 (1.7) | 687 (1.6) |
| **Education: n (%)** [c] | | | | | | | | | | |
| Primary school / None | 7672 (17.6) | 11,145 (25.5) | 13,231 (30.3) | 15,983 (36.6) | 19,255 (44.1) [#] | 6202 (13.9) | 9001 (21.1) | 13,783 (29.3) | 15,817 (39.4) | 22,483 (51.6) [#] |
| Technical school | 12,595 (28.9) | 11,715 (26.8) | 11,100 (25.4) | 10,360 (23.7) | 9382 (21.5) | 11,473 (25.7) | 11,070 (25.9) | 12,496 (26.5) | 10,131 (25.2) | 9982 (22.9) |
| Secondary school | 7657 (17.5) | 7859 (18.0) | 8089 (18.5) | 7535 (17.3) | 6764 (15.5) | 9609 (21.5) | 8567 (20.0) | 8434 (17.9) | 6199 (15.4) | 5095 (11.7) |
| University / longer | 12,684 (29.1) | 10,758 (24.6) | 9441 (21.6) | 8206 (18.8) | 6577 (15.1) | 15,066 (33.7) | 11,874 (27.8) | 9952 (21.1) | 6246 (15.5) | 4528 (10.4) |
| Missing [e] | 3047 (7.0) | 2178 (5.0) | 1795 (4.1) | 1571 (3.6) | 1677 (3.8) | 2317 (5.2) | 2233 (5.2) | 2445 (5.2) | 1785 (4.4) | 1488 (3.4) |
| **Energy: median (IQR)** | | | | | | | | | | |
| kcal/day | 1861 (667) | 1891 (683) | 1907 (680) | 1907 (696) | 1901 (714) | 1895 (683) | 1919 (677) | 1904 (677) | 1879 (687) | 1866 (714) |
| **Age at first period** | | | | | | | | | | |
| Mean (SD) | 12.9 (1.5) | 13.0 (1.6) | 13.1 (1.6) | 13.1 (1.6) | 13.2 (1.6) [#] | 13.1 (1.5) | 13.1 (1.5) | 13.1 (1.6) | 13.1 (1.6) | 13.0 (1.7) [#] |
| Missing: n (%) [e] | 710 (1.6) | 704 (1.6) | 696 (1.6) | 662 (1.5) | 750 (1.7) ^ | 654 (1.5) | 661 (1.5) | 760 (1.6) | 684 (1.7) | 763 (1.8) * |
| **Menopause: n (%)** [c] | | | | | | | | | | |
| Pre-menopausal | 21,109 (48.4) | 17,282 (39.6) | 14,940 (34.2) | 12,640 (29.0) | 9929 (22.7) [#] | 23,393 (52.4) | 17,254 (40.4) | 14,745 (31.3) | 10,727 (26.7) | 9781 (22.4) [#] |
| Peri-menopausal | 6590 (15.1) | 6970 (16.0) | 6730 (15.4) | 6482 (14.8) | 5550 (12.7) | 6404 (14.3) | 6509 (15.2) | 7238 (15.4) | 5878 (14.6) | 6293 (14.4) |
| Post-menopausal | 15,956 (36.6) | 19,403 (44.4) | 21,986 (50.4) | 24,533 (56.2) | 28,176 (64.5) | 14,870 (33.3) | 18,982 (44.4) | 25,127 (53.3) | 23,573 (58.7) | 27,502 (63.1) |
| **Age menopause: n (%)** [c] | | | | | | | | | | |
| <46 years | 2868 (18.0) | 3328 (17.2) | 3908 (17.8) | 4432 (18.1) | 5823 (20.7) [#] | 2538 (17.1) | 3102 (16.3) | 4321 (17.2) | 4541 (19.3) | 5857 (21.3) [#] |
| 46 to <52 years | 6366 (39.9) | 7616 (39.3) | 8677 (39.5) | 9783 (39.9) | 11,170 (39.6) | 5698 (38.3) | 7371 (38.8) | 9944 (39.6) | 9422 (40.0) | 11,177 (40.6) |
| ≥52 years | 3567 (22.4) | 4643 (23.9) | 5209 (23.7) | 5958 (24.3) | 6903 (24.5) | 3045 (20.5) | 4252 (22.4) | 5989 (23.8) | 5764 (24.5) | 7230 (26.3) |
| Age unknown [f] | 3155 (19.8) | 3816 (19.7) | 4192 (19.1) | 4360 (17.8) | 4280 (15.2) | 3589 (24.1) | 4257 (22.4) | 4873 (19.4) | 3846 (16.3) | 3238 (11.8) |
| Mean (SD) | 48.5 (5.2) | 48.7 (4.9) | 48.7 (4.9) | 48.7 (4.9) | 48.5 (5.1) ^ | 48.5 (5.0) | 48.7 (4.9) | 48.7 (5.0) | 48.6 (5.0) | 48.5 (5.1) ^ |

| Characteristics | ABSI | | | | | Waist circumference | | | | |
|---|---|---|---|---|---|---|---|---|---|---|
| | Q1 | Q2 | Q3 | Q4 | Q5 | Q1 | Q2 | Q3 | Q4 | Q5 |
| **Oral contraceptives: n (%) [c]** | | | | | | | | | | |
| Never user | 12,818 (29.4) | 15,056 (34.5) | 16,833 (38.6) | 18,843 (43.2) | 21,770 (49.9) [#] | 12,207 (27.3) | 14,004 (32.8) | 18,455 (39.2) | 18,147 (45.2) | 22,507 (51.6) [#] |
| Former user | 25,389 (58.2) | 24,795 (56.8) | 23,820 (54.6) | 22,652 (51.9) | 20,145 (46.1) | 26,630 (59.6) | 25,007 (58.5) | 25,664 (54.5) | 19,990 (49.8) | 19,510 (44.8) |
| Current user | 5039 (11.5) | 3392 (7.8) | 2614 (6.0) | 1788 (4.1) | 1371 (3.1) | 5474 (12.3) | 3343 (7.8) | 2593 (5.5) | 1639 (4.1) | 1155 (2.7) |
| Missing [e] | 409 (0.9) | 412 (0.9) | 389 (0.9) | 372 (0.9) | 369 (0.8) | 356 (0.8) | 391 (0.9) | 398 (0.8) | 402 (1.0) | 404 (0.9) |
| **HRT: n (%) [c]** | | | | | | | | | | |
| Never user | 30,300 (69.4) | 28,769 (65.9) | 28,866 (66.1) | 29,247 (67.0) | 30,280 (69.4) [#] | 32,048 (71.7) | 28,408 (66.5) | 30,263 (64.2) | 26,552 (66.1) | 30,191 (69.3) [#] |
| Former user | 2972 (6.8) | 3399 (7.8) | 3830 (8.8) | 4171 (9.6) | 4463 (10.2) | 2456 (5.5) | 3282 (7.7) | 4457 (9.5) | 4130 (10.3) | 4510 (10.3) |
| Current user | 6461 (14.8) | 7268 (16.6) | 7328 (16.8) | 7178 (16.4) | 6315 (14.5) | 6456 (14.5) | 7323 (17.1) | 8539 (18.1) | 6511 (16.2) | 5721 (13.1) |
| Missing [f] | 3922 (9.0) | 4219 (9.7) | 3632 (8.3) | 3059 (7.0) | 2597 (5.9) | 3707 (8.3) | 3732 (8.7) | 3851 (8.2) | 2985 (7.4) | 3154 (7.2) |
| **Parity: n (%) [c]** | | | | | | | | | | |
| Nulliparous | 11,069 (25.4) | 7697 (17.6) | 6448 (14.8) | 5657 (13.0) | 5676 (13.0) [#] | 12613 (28.2) | 7820 (18.3) | 6623 (14.1) | 4895 (12.2) | 4596 (10.5) [#] |
| One at <25 years | 2402 (5.5) | 2441 (5.6) | 2290 (5.2) | 2216 (5.1) | 1898 (4.3) | 2455 (5.5) | 2190 (5.1) | 2486 (5.3) | 2056 (5.1) | 2060 (4.7) |
| One at ≥25 years | 4660 (10.7) | 4826 (11.1) | 4523 (10.4) | 4280 (9.8) | 3863 (8.8) | 5302 (11.9) | 4828 (11.3) | 4716 (10.0) | 3783 (9.4) | 3523 (8.1) |
| One unknown age [g] | 28 (0.1) | 27 (0.1) | 36 (0.1) | 30 (0.1) | 29 (0.1) | 33 (0.1) | 30 (0.1) | 33 (0.1) | 27 (0.1) | 27 (0.1) |
| Two at <25 years | 7402 (17.0) | 8542 (19.6) | 8444 (19.3) | 8023 (18.4) | 7195 (16.5) | 6900 (15.4) | 7725 (18.1) | 9155 (19.4) | 7828 (19.5) | 7998 (18.4) |
| Two at ≥25 years | 7920 (18.1) | 8688 (19.9) | 8982 (20.6) | 8837 (20.2) | 8029 (18.4) | 8268 (18.5) | 9175 (21.5) | 9882 (21.0) | 7724 (19.2) | 7407 (17.0) |
| Two unknown age [g] | 38 (0.1) | 37 (0.1) | 45 (0.1) | 45 (0.1) | 39 (0.1) | 30 (0.1) | 43 (0.1) | 49 (0.1) | 27 (0.1) | 55 (0.1) |
| ≥3 at <25 years | 4988 (11.4) | 5746 (13.2) | 6591 (15.1) | 7634 (17.5) | 8699 (19.9) | 3887 (8.7) | 5211 (12.2) | 7077 (15.0) | 7243 (18.0) | 10,240 (23.5) |
| ≥3 at ≥25 years | 2808 (6.4) | 3389 (7.8) | 4048 (9.3) | 4687 (10.7) | 5567 (12.8) | 2784 (6.2) | 3513 (8.2) | 4612 (9.8) | 4292 (10.7) | 5298 (12.2) |
| ≥3 unknown age [g] | 26 (0.1) | 25 (0.1) | 20 (0.0) | 31 (0.1) | 40 (0.1) | 14 (0.0) | 24 (0.1) | 32 (0.1) | 33 (0.1) | 39 (0.1) |
| Missing [g] | 2314 (5.3) | 2237 (5.1) | 2229 (5.1) | 2215 (5.1) | 2620 (6.0) | 2381 (5.3) | 2186 (5.1) | 2445 (5.2) | 2270 (5.6) | 2333 (5.4) |
| **Breastfeeding: n (%) [c]** | | | | | | | | | | |
| Never | 13,900 (31.8) | 11,414 (26.1) | 10,668 (24.4) | 10,523 (24.1) | 10,359 (23.7) [#] | 15,786 (35.3) | 11,371 (26.6) | 11,132 (23.6) | 9018 (22.4) | 9557 (21.9) [#] |
| <6 months | 12,265 (28.1) | 1,3965 (32.0) | 14,316 (32.8) | 14,086 (32.3) | 12,986 (29.7) | 12,347 (27.6) | 13,514 (31.6) | 15,753 (33.4) | 12,882 (32.1) | 13,122 (30.1) |
| ≥6 months | 13,397 (30.7) | 14,649 (33.6) | 15,236 (34.9) | 15,687 (35.9) | 16,475 (37.7) | 12,358 (27.7) | 14,240 (33.3) | 16,390 (34.8) | 14,933 (37.2) | 17,523 (40.2) |
| Duration unknown [h] | 90 (0.2) | 171 (0.4) | 175 (0.4) | 210 (0.5) | 199 (0.5) | 190 (0.4) | 172 (0.4) | 180 (0.4) | 170 (0.4) | 133 (0.3) |
| Missing [h] | 4003 (9.2) | 3456 (7.9) | 3261 (7.5) | 3149 (7.2) | 3636 (8.3) | 3986 (8.9) | 3448 (8.1) | 3655 (7.8) | 3175 (7.9) | 3241 (7.4) |

| Characteristics | ABSI | | | | | Waist circumference | | | | |
|---|---|---|---|---|---|---|---|---|---|---|
| | Q1 | Q2 | Q3 | Q4 | Q5 | Q1 | Q2 | Q3 | Q4 | Q5 |
| **Country: n (%)** [c] | | | | | | | | | | |
| France | 1937 (4.4) | 3437 (7.9) | 4467 (10.2) | 4864 (11.1) | 4417 (10.1) [#] | 5292 (11.8) | 4793 (11.2) | 4271 (9.1) | 2716 (6.8) | 2050 (4.7) [#] |
| Italy | 4608 (10.6) | 6206 (14.2) | 6582 (15.1) | 6680 (15.3) | 5905 (13.5) | 5363 (12.0) | 5781 (13.5) | 6820 (14.5) | 6159 (15.3) | 5858 (13.4) |
| Spain | 1690 (3.9) | 3120 (7.1) | 4420 (10.1) | 6150 (14.1) | 9168 (21.0) | 1031 (2.3) | 2431 (5.7) | 4583 (9.7) | 6071 (15.1) | 10,432 (23.9) |
| United Kingdom | 18,608 (42.6) | 10,788 (24.7) | 7825 (17.9) | 5981 (13.7) | 4880 (11.2) | 16,571 (37.1) | 11,609 (27.2) | 9342 (19.8) | 6059 (15.1) | 4501 (10.3) |
| Netherlands | 4290 (9.8) | 5133 (11.8) | 5630 (12.9) | 5840 (13.4) | 5807 (13.3) | 4214 (9.4) | 4858 (11.4) | 6249 (13.3) | 5815 (14.5) | 5564 (12.8) |
| Germany | 4578 (10.5) | 6078 (13.9) | 5920 (13.6) | 5630 (12.9) | 4934 (11.3) | 4889 (10.9) | 5086 (11.9) | 5878 (12.5) | 5157 (12.8) | 6130 (14.1) |
| Denmark | 4261 (9.8) | 3627 (8.3) | 2684 (6.1) | 2019 (4.6) | 1451 (3.3) | 3630 (8.1) | 3045 (7.1) | 3138 (6.7) | 2227 (5.5) | 2002 (4.6) |
| Sweden | 3683 (8.4) | 5266 (12.1) | 6128 (14.0) | 6491 (14.9) | 7093 (16.2) | 3677 (8.2) | 5142 (12.0) | 6829 (14.5) | 5974 (14.9) | 7039 (16.2) |

**ABSI** – a body shape index; **BMI** – body mass index; **ER+/-** – oestrogen receptor status; **HC** – hip circumference; **HER2** – human epidermal receptor 2 status; **HI** – hip index; **n** – number; **HRT** – hormone replacement therapy; **IQR** – interquartile range; **PR+/-** – progesterone receptor status; **Q1-Q5** – study-specific quintiles (cut-offs: 68.843, 71.525, 73.919, 76.884 for ABSI; 70, 75, 81, 88.5 cm for WC); **SD** – standard deviation; **WC** – waist circumference.

Missing values were either imputed with the median category or value, within subgroups by country and menopausal status at the anthropometric assessment, or were used as a separate category as follows:

[a] – mean (standard deviation);

[b] – number (percent form cases with available ERPR status (for ERPR) or with available ERPRHER2 status (for HER2) per quintile);

[c] – number (percent form total cases per quintile (for ERPR and HER2) or total women per quintile for other variables);

[d] – missing quit time was imputed with the median category within former smokers; missing number of cigarettes per day was imputed with the median category within current smokers; missing smoking status was imputed with the median category overall;

[e] – missing physical activity, education, and oral contraceptives use were imputed with the median category, and missing age at the first period was imputed with the median value;

[f] – menopause at unknown age and missing HRT use were used as separate categories;

[g] – missing age at the first live birth was imputed with the median category within the subgroup of women with the corresponding number of children; missing parity was used as a separate category;

[h] – missing breastfeeding duration was imputed with the median category within breastfeeding women; missing breastfeeding information was used as a separate category.

**p-values**: ^ ≥0.05; * <0.05; [#] <0.0001 from ANOVA test for continuous variables and $\chi^2$ test for categorical variables, comparing quintile categories.

**Supplementary Table S2 Characteristics of breast cancer cases according to hormone receptor status**

| Characteristics | ER+PR+ | ER+PR- | ER-PR- [a] | ER+/-PR+/- | ER?PR? [b] |
|---|---|---|---|---|---|
| Cases: n (%) | 3101 | 726 | 759 | 4692 | 4319 |
| Follow-up: years: mean (SD) | 8.0 (4.0) | 7.7 (4.1) | 7.6 (4.1) * | 7.8 (4.1) | 7.7 (5.1) ^ |
| Date diagnosis: year-month | 2005-1 (6.1) | 2004-7 (6.3) | 2004-5 (5.8) * | 2004-9 (6.1) | 2003-1 (8.9) # |
| Age diagnosis: years: mean (SD) | 61.7 (8.5) | 63.0 (7.7) | 61.2 (8.9) ^ | 61.7 (8.5) | 62.8 (9.7) # |
| Age baseline: years: mean (SD) | 53.2 (8.2) | 54.8 (7.2) | 53.2 (8.4) ^ | 53.4 (8.1) | 54.7 (8.5) # |
| **Grade**: n (%) | | | | | |
| Well differentiated [c] | 414 (21.2) | 82 (19.3) | 20 ( 4.8) # | 531 (18.6) | 115 (17.9) ^ |
| Moderately differentiated [c] | 1021 (52.2) | 202 (47.6) | 82 (19.8) | 1330 (46.5) | 296 (46.0) |
| Poorly differentiated [c] | 520 (26.6) | 140 (33.0) | 313 (75.4) | 1001 (35.0) | 233 (36.2) |
| Missing [d] | 1146 (37.0) | 302 (41.6) | 344 (45.3) # | 1830 (39) | 3675 (85.1) # |
| **Stage**: n (%) | | | | | |
| Localised [c] | 1304 (70.2) | 307 (71.4) | 270 (61.2) * | 1948 (69.1) | 754 (64.9) * |
| Metastatic [c] | 553 (29.8) | 123 (28.6) | 171 (38.8) | 870 (30.9) | 407 (35.1) |
| Missing [d] | 1244 (40.1) | 296 (40.8) | 318 (41.9) ^ | 1874 (39.9) | 3158 (73.1) # |
| **Anthropometry**: mean (SD) | | | | | |
| Height: cm | 162.1 (6.6) | 162.1 (6.7) | 162.1 (6.3) ^ | 162.1 (6.5) | 163.3 (6.4) # |
| Weight: kg | 67.6 (12.4) | 66.3 (11.2) | 66.5 (11.4) * | 67.1 (12.0) | 67.8 (11.8) * |
| BMI: kg/m$^2$ | 25.7 (4.6) | 25.3 (4.3) | 25.3 (4.4) * | 25.6 (4.5) | 25.4 (4.3) ^ |
| WC: cm | 81.2 (11.4) | 80.1 (10.5) | 80.0 (10.6) * | 80.8 (11.2) | 80.0 (10.9) * |
| HC: cm | 102.0 (9.3) | 101.1 (8.4) | 101.2 (8.6) * | 101.7 (9.1) | 100.8 (9.0) # |
| ABSI | 73.4 (4.8) | 73.3 (5.0) | 73.1 (4.8) ^ | 73.3 (4.8) | 72.6 (5.1) # |
| HI | 65.1 (2.7) | 65.1 (2.6) | 65.1 (2.6) ^ | 65.1 (2.6) | 64.3 (2.7) # |
| **Smoking: n (%)** | | | | | |
| Never smoker | 1757 (56.7) | 391 (53.9) | 419 (55.2) ^ | 2628 (56.0) | 2118 (49.0) # |
| Former: quit ≥15 years | 350 (11.3) | 88 (12.1) | 79 (10.4) | 529 (11.3) | 625 (14.5) |
| Former: quit <15 years | 333 (10.7) | 88 (12.1) | 99 (13.0) | 535 (11.4) | 528 (12.2) |
| Former: time missing | 22 (0.7) | 5 (0.7) | 6 (0.8) | 33 (0.7) | 49 (1.1) |
| Current: ≤10 cig./day | 243 (7.8) | 65 (9.0) | 63 (8.3) | 380 (8.1) | 378 (8.8) |
| Current: >10 cig./day | 357 (11.5) | 76 (10.5) | 83 (10.9) | 524 (11.2) | 550 (12.7) |
| Current: cig. missing | 10 (0.3) | 2 (0.3) | 2 (0.3) | 14 (0.3) | 26 (0.6) |
| Missing | 29 (0.9) | 11 (1.5) | 8 (1.1) | 49 (1.0) | 45 (1.0) |
| **Alcohol: n (%)** | | | | | |
| None | 441 (14.2) | 115 (15.8) | 87 (11.5) ^ | 656 (14.0) | 515 (11.9) # |
| Light: <4 g/day | 968 (31.2) | 211 (29.1) | 249 (32.8) | 1463 (31.2) | 1302 (30.1) |
| Moderate: 4 to <16 g/day | 969 (31.2) | 231 (31.8) | 250 (32.9) | 1484 (31.6) | 1663 (38.5) |
| Heavy: ≥16 g/day | 723 (23.3) | 169 (23.3) | 173 (22.8) | 1089 (23.2) | 839 (19.4) |
| **Physical activity: n (%)** | | | | | |
| Inactive | 717 (23.1) | 153 (21.1) | 154 (20.3) ^ | 1056 (22.5) | 936 (21.7) * |
| Moderately inactive | 1130 (36.4) | 252 (34.7) | 274 (36.1) | 1687 (36.0) | 1542 (35.7) |
| Moderately active | 692 (22.3) | 176 (24.2) | 181 (23.8) | 1070 (22.8) | 960 (22.2) |
| Active | 533 (17.2) | 140 (19.3) | 145 (19.1) | 839 (17.9) | 795 (18.4) |
| Missing | 29 (0.9) | 5 (0.7) | 5 (0.7) | 40 (0.9) | 86 (2.0) |
| **Education: n (%)** | | | | | |
| Primary school / None | 971 (31.3) | 228 (31.4) | 235 (31.0) ^ | 1475 (31.4) | 1160 (26.9) # |
| Technical school | 715 (23.1) | 154 (21.2) | 187 (24.6) | 1074 (22.9) | 1380 (32.0) |
| Secondary school | 628 (20.3) | 155 (21.3) | 159 (20.9) | 971 (20.7) | 559 (12.9) |
| University / longer | 672 (21.7) | 170 (23.4) | 147 (19.4) | 1006 (21.4) | 907 (21.0) |
| Missing | 115 (3.7) | 19 (2.6) | 31 (4.1) | 166 (3.5) | 313 (7.2) |

| Characteristics | ER+PR+ | ER+PR- | ER-PR- | ER+/-PR+/- | ER?PR? |
|---|---|---|---|---|---|
| **Energy: median (IQR)** | | | | | |
| kcal/day | 1926 (694) | 1937 (687) | 1951 (648) ^ | 1932 (684) | 1894 (644) * |
| **Age at first period** | | | | | |
| Mean (SD) | 13.0 (1.5) | 13.1 (1.6) | 13.1 (1.6) ^ | 13.0 (1.5) | 13.2 (1.6) # |
| Missing: n (%) | 33 (1.1) | 15 (2.1) | 11 (1.4) ^ | 59 (1.3) | 131 (3.0) # |
| **Menopause: n (%)** | | | | | |
| Pre-menopausal | 908 (29.3) | 153 (21.1) | 209 (27.5) * | 1322 (28.2) | 856 (19.8) # |
| Peri-menopausal | 502 (16.2) | 127 (17.5) | 120 (15.8) | 764 (16.3) | 801 (18.5) |
| Post-menopausal | 1691 (54.5) | 446 (61.4) | 430 (56.7) | 2606 (55.5) | 2662 (61.6) |
| **Age menopause: n (%)** | | | | | |
| <46 years | 253 (15.0) | 48 (10.8) | 76 (17.7) ^ | 381 (14.6) | 414 (15.6) ^ |
| 46 to <52 years | 641 (37.9) | 167 (37.4) | 165 (38.4) | 987 (37.9) | 986 (37.0) |
| ≥52 years | 413 (24.4) | 130 (29.1) | 95 (22.1) | 645 (24.8) | 722 (27.1) |
| Age unknown | 384 (22.7) | 101 (22.6) | 94 (21.9) | 593 (22.8) | 540 (20.3) |
| Mean (SD) | 49.1 (4.7) | 49.7 (5.0) | 48.5 (5.1) ^ | 49.1 (4.8) | 49.1 (5.0) ^ |
| **Oral contraceptives: n (%)** | | | | | |
| Never user | 1267 (40.9) | 318 (43.8) | 281 (37.0) ^ | 1910 (40.7) | 1760 (40.8) # |
| Former user | 1686 (54.4) | 378 (52.1) | 445 (58.6) | 2564 (54.6) | 2333 (54.0) |
| Current user | 130 (4.2) | 28 (3.9) | 30 (4.0) | 195 (4.2) | 155 (3.6) |
| Missing | 18 (0.6) | 2 (0.3) | 3 (0.4) | 23 (0.5) | 71 (1.6) |
| **HRT: n (%)** | | | | | |
| Never user | 1946 (62.8) | 465 (64.0) | 463 (61.0) ^ | 2943 (62.7) | 2286 (52.9) # |
| Former user | 295 (9.5) | 77 (10.6) | 81 (10.7) | 460 (9.8) | 408 (9.4) |
| Current user | 717 (23.1) | 163 (22.5) | 183 (24.1) | 1085 (23.1) | 1097 (25.4) |
| Missing | 143 (4.6) | 21 (2.9) | 32 (4.2) | 204 (4.3) | 528 (12.2) |
| **Parity: n (%)** | | | | | |
| Nulliparous | 463 (14.9) | 107 (14.7) | 103 (13.6) * | 686 (14.6) | 684 (15.8) ^ |
| One at <25 years | 148 (4.8) | 32 (4.4) | 49 (6.5) | 234 (5.0) | 212 (4.9) |
| One at ≥25 years | 358 (11.5) | 102 (14.0) | 70 (9.2) | 542 (11.6) | 467 (10.8) |
| One unknown age | 0 (0.0) | 1 (0.1) | 0 (0.0) | 1 (0.0) | 3 (0.1) |
| Two at <25 years | 581 (18.7) | 114 (15.7) | 168 (22.1) | 883 (18.8) | 794 (18.4) |
| Two at ≥25 years | 703 (22.7) | 155 (21.3) | 134 (17.7) | 1018 (21.7) | 923 (21.4) |
| Two unknown age | 5 (0.2) | 0 (0.0) | 0 (0.0) | 5 (0.1) | 7 (0.2) |
| ≥3 at <25 years | 415 (13.4) | 96 (13.2) | 126 (16.6) | 648 (13.8) | 632 (14.6) |
| ≥3 at ≥25 years | 300 (9.7) | 79 (10.9) | 76 (10.0) | 470 (10.0) | 378 (8.8) |
| ≥3 unknown age | 1 (0.0) | 0 (0.0) | 1 (0.1) | 2 (0.0) | 4 (0.1) |
| Missing | 127 (4.1) | 40 (5.5) | 32 (4.2) | 203 (4.3) | 215 (5.0) |
| **Breastfeeding: n (%)** | | | | | |
| Never | 823 (26.5) | 198 (27.3) | 194 (25.6) ^ | 1237 (26.4) | 1048 (24.3) # |
| <6 months | 1032 (33.3) | 240 (33.1) | 251 (33.1) | 1561 (33.3) | 1224 (28.3) |
| ≥6 months | 1058 (34.1) | 236 (32.5) | 266 (35.0) | 1598 (34.1) | 1687 (39.1) |
| Duration unknown | 21 (0.7) | 6 (0.8) | 3 (0.4) | 34 (0.7) | 15 (0.3) |
| Missing | 167 (5.4) | 46 (6.3) | 45 (5.9) | 262 (5.6) | 345 (8.0) |
| **Country: n (%)** | | | | | |
| France | 462 (14.9) | 123 (16.9) | 109 (14.4) # | 711 (15.2) | 116 (2.7) # |
| Italy | 647 (20.9) | 175 (24.1) | 128 (16.9) | 988 (21.1) | 196 (4.5) |
| Spain | 292 (9.4) | 62 (8.5) | 73 (9.6) | 442 (9.4) | 205 (4.7) |
| United Kingdom | 310 (10.0) | 51 (7.0) | 84 (11.1) | 449 (9.6) | 1325 (30.7) |
| Netherlands | 424 (13.7) | 121 (16.7) | 92 (12.1) | 642 (13.7) | 373 (8.6) |
| Germany | 494 (15.9) | 62 (8.5) | 109 (14.4) | 679 (14.5) | 130 (3.0) |
| Denmark | - | - | - | - | 890 (20.6) |
| Sweden | 472 (15.2) | 132 (18.2) | 164 (21.6) | 781 (16.6) | 1084 (25.1) |

**ABSI** – a body shape index; **BMI** – body mass index; **ER+/-/?** – oestrogen receptor status (positive/ negative/ unknown); **HC** – hip circumference; **HI** – hip index; **HRT** – hormone replacement therapy; **IQR** – interquartile range; **n (%)** – number (percent from total per column); **PR+/-/?** – progesterone receptor status (positive/ negative/ unknown); **SD** – standard deviation; **WC** – waist circumference.

Missing values were either imputed with the median category or value, within subgroups by country and menopausal status at the anthropometric assessment, or were used as a separate category (see details in the legend of Supplementary Table S1).

[a] – p-value for comparisons between breast cancer subtypes;

[b] – p-value for comparisons between breast cancer cases with available and missing ERPR status;

[c] – comparisons for women with available grade or stage;

[d] – comparisons for missing vs available grade or stage.

**p-values**: ^ ≥0.05; * <0.05; [#] <0.0001 from ANOVA test for continuous variables and $\chi^2$ test for categorical variables.

**Supplementary Table S3 Breast cancer risk according to allometric anthropometric index quintiles**

| | ABSI | | | HI | | | BMI | | |
|---|---|---|---|---|---|---|---|---|---|
| | Cases | HR (95% CI) | p-value | Cases | HR (95% CI) | p-value | Cases | HR (95% CI) | p-value |
| **Overall** | | | | | | | | | |
| Q1 | 1736 | reference | | 1939 | reference | | 1554 | reference | |
| Q2 | 1879 | 1.025 (0.959 to 1.096) | 0.463 | 1779 | 0.960 (0.899 to 1.025) | 0.222 | 1822 | **1.091 (1.019 to 1.168)** | **0.013** |
| Q3 | 1761 | 0.939 (0.877 to 1.006) | 0.073 | 1778 | 0.982 (0.919 to 1.050) | 0.592 | 1903 | **1.139 (1.063 to 1.220)** | **<0.001** |
| Q4 | 1835 | 0.972 (0.907 to 1.042) | 0.424 | 1725 | 0.977 (0.912 to 1.046) | 0.501 | 1884 | **1.153 (1.074 to 1.237)** | **<0.001** |
| Q5 | 1800 | 0.974 (0.907 to 1.046) | 0.469 | 1790 | 1.060 (0.989 to 1.136) | 0.098 | 1848 | **1.241 (1.153 to 1.335)** | **<0.001** |
| $p_{non\text{-}linearity}$ | | 0.363 | | | 0.331 | | | 0.427 | |
| **Pre-MP at baseline** | | | | | | | | | |
| Q1 | 549 | reference | | 418 | reference | | 559 | reference | |
| Q2 | 501 | 1.002 (0.885 to 1.135) | 0.970 | 432 | 0.998 (0.871 to 1.143) | 0.974 | 519 | 1.042 (0.922 to 1.177) | 0.510 |
| Q3 | 424 | 0.928 (0.813 to 1.060) | 0.273 | 450 | 1.066 (0.930 to 1.222) | 0.360 | 449 | 1.056 (0.928 to 1.202) | 0.408 |
| Q4 | 406 | 1.029 (0.897 to 1.179) | 0.686 | 456 | 1.093 (0.952 to 1.254) | 0.208 | 337 | 0.936 (0.811 to 1.080) | 0.365 |
| Q5 | 298 | 0.965 (0.828 to 1.124) | 0.645 | 422 | 1.056 (0.915 to 1.219) | 0.459 | 314 | 0.984 (0.845 to 1.145) | 0.832 |
| $p_{non\text{-}linearity}$ | | 0.781 | | | 0.301 | | | 0.622 | |
| **Post-MP at baseline** | | | | | | | | | |
| Q1 | 854 | reference | | 1077 | reference | | 707 | reference | |
| Q2 | 1034 | 1.032 (0.941 to 1.131) | 0.507 | 1063 | 0.991 (0.909 to 1.080) | 0.832 | 971 | **1.144 (1.037 to 1.261)** | **0.007** |
| Q3 | 1025 | 0.925 (0.842 to 1.016) | 0.102 | 1061 | 1.001 (0.917 to 1.092) | 0.986 | 1141 | **1.230 (1.118 to 1.354)** | **<0.001** |
| Q4 | 1113 | 0.926 (0.844 to 1.017) | 0.106 | 977 | 0.952 (0.870 to 1.041) | 0.281 | 1238 | **1.289 (1.171 to 1.419)** | **<0.001** |
| Q5 | 1242 | 0.950 (0.865 to 1.043) | 0.278 | 1090 | **1.101 (1.006 to 1.204)** | **0.037** | 1211 | **1.386 (1.255 to 1.531)** | **<0.001** |
| $p_{non\text{-}linearity}$ | | 0.247 | | | 0.192 | | | 0.104 | |
| **Diagnosis at age<55 years** | | | | | | | | | |
| Q1 | 495 | reference | | 414 | reference | | 523 | reference | |
| Q2 | 450 | 1.018 (0.893 to 1.161) | 0.788 | 365 | 0.926 (0.802 to 1.069) | 0.293 | 492 | 1.074 (0.948 to 1.218) | 0.262 |
| Q3 | 384 | 0.983 (0.855 to 1.130) | 0.808 | 407 | 1.070 (0.929 to 1.234) | 0.347 | 389 | 1.012 (0.883 to 1.161) | 0.859 |
| Q4 | 376 | 1.119 (0.969 to 1.291) | 0.125 | 405 | 1.076 (0.931 to 1.243) | 0.320 | 293 | 0.901 (0.774 to 1.049) | 0.179 |
| Q5 | 263 | 0.991 (0.843 to 1.165) | 0.911 | 377 | 1.055 (0.908 to 1.227) | 0.482 | 271 | 0.953 (0.812 to 1.119) | 0.557 |
| $p_{non\text{-}linearity}$ | | 0.822 | | | 0.201 | | | 0.298 | |

| | ABSI | | | HI | | | BMI | | |
|---|---|---|---|---|---|---|---|---|---|
| | Cases | HR (95% CI) | p-value | Cases | HR (95% CI) | p-value | Cases | HR (95% CI) | p-value |
| **Diagnosis at age≥55 years** | | | | | | | | | |
| Q1 | 1241 | reference | | 1525 | reference | | 1031 | reference | |
| Q2 | 1429 | 1.022 (0.946 to 1.104) | 0.589 | 1414 | 0.968 (0.900 to 1.043) | 0.395 | 1330 | **1.112 (1.025 to 1.208)** | **0.011** |
| Q3 | 1377 | **0.922 (0.852 to 0.998)** | **0.045** | 1371 | 0.958 (0.888 to 1.034) | 0.269 | 1514 | **1.201 (1.108 to 1.303)** | **<0.001** |
| Q4 | 1459 | 0.933 (0.862 to 1.011) | 0.089 | 1320 | 0.950 (0.880 to 1.027) | 0.197 | 1591 | **1.246 (1.149 to 1.352)** | **<0.001** |
| Q5 | 1537 | 0.960 (0.886 to 1.041) | 0.324 | 1413 | 1.060 (0.980 to 1.146) | 0.146 | 1577 | **1.344 (1.235 to 1.463)** | **<0.001** |
| $p_{\text{non-linearity}}$ | | 0.151 | | | 0.174 | | | 0.076 | |
| **ER+PR+** | | | | | | | | | |
| Q1 | 498 | reference | | 513 | reference | | 524 | reference | |
| Q2 | 593 | 0.950 (0.842 to 1.072) | 0.407 | 586 | 0.961 (0.853 to 1.082) | 0.510 | 586 | 1.065 (0.945 to 1.200) | 0.301 |
| Q3 | 633 | 0.908 (0.805 to 1.025) | 0.118 | 618 | 0.957 (0.850 to 1.078) | 0.467 | 662 | **1.196 (1.063 to 1.347)** | **0.003** |
| Q4 | 710 | 0.959 (0.851 to 1.081) | 0.491 | 668 | 0.997 (0.886 to 1.122) | 0.964 | 639 | **1.172 (1.037 to 1.325)** | **0.011** |
| Q5 | 667 | 0.906 (0.801 to 1.026) | 0.119 | 716 | 1.048 (0.931 to 1.181) | 0.435 | 690 | **1.346 (1.188 to 1.526)** | **<0.001** |
| $p_{\text{non-linearity}}$ | | 0.906 | | | 0.271 | | | 0.585 | |
| **ER+PR-** | | | | | | | | | |
| Q1 | 114 | reference | | 113 | reference | | 131 | reference | |
| Q2 | 163 | 1.046 (0.821 to 1.333) | 0.715 | 141 | 0.998 (0.778 to 1.280) | 0.986 | 141 | 1.009 (0.793 to 1.285) | 0.939 |
| Q3 | 154 | 0.859 (0.670 to 1.100) | 0.227 | 161 | 1.049 (0.822 to 1.340) | 0.699 | 167 | 1.162 (0.917 to 1.472) | 0.213 |
| Q4 | 145 | **0.752 (0.584 to 0.969)** | **0.028** | 148 | 0.916 (0.713 to 1.177) | 0.493 | 152 | 1.074 (0.838 to 1.376) | 0.573 |
| Q5 | 150 | **0.760 (0.588 to 0.983)** | **0.037** | 163 | 0.967 (0.753 to 1.243) | 0.796 | 135 | 1.040 (0.798 to 1.356) | 0.770 |
| $p_{\text{non-linearity}}$ | | **0.020** | | | 0.616 | | | 0.777 | |
| **ER-PR-** | | | | | | | | | |
| Q1 | 135 | reference | | 134 | reference | | 142 | reference | |
| Q2 | 163 | 0.983 (0.779 to 1.240) | 0.885 | 142 | 0.905 (0.713 to 1.148) | 0.410 | 157 | 1.027 (0.816 to 1.293) | 0.820 |
| Q3 | 157 | 0.853 (0.673 to 1.082) | 0.190 | 157 | 0.952 (0.753 to 1.203) | 0.682 | 150 | 0.960 (0.757 to 1.217) | 0.736 |
| Q4 | 141 | **0.733 (0.573 to 0.938)** | **0.013** | 136 | 0.802 (0.628 to 1.024) | 0.077 | 158 | 1.009 (0.793 to 1.285) | 0.940 |
| Q5 | 163 | 0.832 (0.651 to 1.064) | 0.143 | 190 | 1.101 (0.873 to 1.389) | 0.415 | 152 | 1.012 (0.785 to 1.304) | 0.929 |
| $p_{\text{non-linearity}}$ | | 0.794 | | | 0.613 | | | 0.791 | |

| | ABSI | | | HI | | | BMI | | |
|---|---|---|---|---|---|---|---|---|---|
| | Cases | HR (95% CI) | p-value | Cases | HR (95% CI) | p-value | Cases | HR (95% CI) | p-value |
| **ER+/-PR-** | | | | | | | | | |
| Q1 | 249 | reference | | 247 | reference | | 273 | reference | |
| Q2 | 326 | 1.013 (0.857 to 1.197) | 0.882 | 283 | 0.949 (0.799 to 1.127) | 0.551 | 298 | 1.019 (0.863 to 1.204) | 0.823 |
| Q3 | 311 | 0.856 (0.721 to 1.016) | 0.076 | 318 | 0.998 (0.843 to 1.182) | 0.983 | 317 | 1.059 (0.895 to 1.251) | 0.505 |
| Q4 | 286 | **0.743 (0.623 to 0.886)** | **<0.001** | **284** | 0.858 (0.720 to 1.022) | 0.085 | 310 | 1.042 (0.877 to 1.238) | 0.641 |
| Q5 | 313 | **0.797 (0.667 to 0.951)** | **0.012** | **353** | 1.035 (0.873 to 1.227) | 0.694 | 287 | 1.027 (0.855 to 1.233) | 0.777 |
| $p_{\text{non-linearity}}$ | | **0.050** | | | 0.427 | | | 0.666 | |

**ABSI** – a body shape index; **CI** – confidence interval; **ER+/-** – oestrogen receptor status; **HI** – hip index; **HR** – hazard ratio; **p-value** – from Wald test for the individual term; **Pre-MP** – pre-menopausal; **Post-MP** – post-menopausal; **PR+/-** – progesterone receptor status; **Q1-Q5** – study-specific quintile categories.

**HR (95% CI)** (compared to the lowest quintile) were derived from Cox proportional hazards models, including quintile categories for ASBI, HI, and BMI as exposures (lowest quintile reference, cut-offs: 68.843, 71.525, 73.919, 76.884 for ABSI; 62.763, 64.240, 65.477, 66.933 for HI; 21.63, 23.54, 25.58, 28.55 kg/m$^2$ for BMI), stratified by age at the anthropometric assessment, country, and categories of menopausal status and, for post-menopausal women, age at menopause (pre-menopausal, peri-menopausal, menopause at <46 years, menopause at 46 to <52 years, menopause at ≥52 years, menopause at unknown age), and adjusted for height (continuous), smoking status and intensity (never smoker, former quit≥15 years, former quit<15 years, current ≤10 cigarettes/day, current >10 cigarettes/day), alcohol consumption (none, <4 g/day, 4 to <16 g/day, ≥16 g/day), physical activity (inactive, moderately inactive, moderately active, active), education (primary/none, technical, secondary, university/longer), hormone replacement therapy use (never, former, current, missing), oral contraceptives use (never, former, current), age at the first period (continuous), parity with age at first live birth (nulliparous, one at <25 years, one at ≥25 years, two at <25 years, two at ≥25 years, ≥3 at <25 years, ≥3 at ≥25 years, missing), breastfeeding with duration (never, <6 months, ≥6 months, missing), and energy intake (log-transformed continuous); $\mathbf{p}_{\textbf{non-linearity}}$ – obtained from likelihood ratio tests comparing the fully adjusted models, including ABSI, HI, and BMI on a linear untransformed scale, with models including restricted cubic splines, individually for each anthropometric index, with knots at the corresponding quintile cut-offs.

**Supplementary Table S4 Correlations between traditional and allometric anthropometric indices**

| | Mean (SD) | WC | WCadjBMI | WCadjWtHt | ABSI | HC | HCadjBMI | HCadjWtHt | HI | BMI |
|---|---|---|---|---|---|---|---|---|---|---|
| **WC** | 79.8 (11.2) | | | | | | | | | |
| **WCadjBMI** | 0 (5.8) | 0.47 | | | | | | | | |
| **WCadjWtHt** | 0 (5.6) | 0.43 | 0.97 | | | | | | | |
| **ABSI** | 72.9 (5.1) | 0.53 | 0.95 | 0.98 | | | | | | |
| **HC** | 100.7 (9.1) | 0.78 | 0.05 | -0.02 | 0.06 | | | | | |
| **HCadjBMI** | 0 (4.7) | 0.12 | 0.22 | 0.13 | 0.10 | 0.53 | | | | |
| **HCadjWtHt** | 0 (4.2) | 0.02 | 0.11 | 0.12 | 0.09 | 0.44 | 0.91 | | | |
| **HI** | 64.8 (2.7) | 0.00 | 0.06 | 0.09 | 0.09 | 0.41 | 0.84 | 0.97 | | |
| **BMI** | 25.3 (4.5) | 0.84 | -0.08 | -0.10 | 0.01 | 0.85 | -0.00 | -0.04 | -0.04 | |
| **Height** | 162.0 (6.7) | 0.06 | 0.32 | 0.12 | 0.05 | 0.14 | 0.47 | 0.09 | -0.06 | -0.12 |

**ABSI** – a body shape index; **BMI** – body mass index; **HC** – hip circumference; **HCadjBMI** – residuals of HC from linear regression on BMI; **HCadjWtHt** – residuals of HC from linear regression on weight and height; **HI** – hip index; **WC** – waist circumference; **WCadjBMI** – residuals of WC from linear regression on BMI; **WCadjWtHt** – residuals of WC from linear regression on weight and height.

Values represent partial Pearson correlation coefficients, calculated in the study dataset, with adjustment for age at the anthropometric assessment (continuous), country, and categories of menopausal status and, for post-menopausal women, age at menopause (pre-menopausal, peri-menopausal, menopause at <46 years, menopause at 46 to <52 years, menopause at ≥52 years, menopause at unknown age).

**Supplementary Table S5 Comparisons of traditional and allometric anthropometric indices with respect to breast cancer risk**

| | **Waist size** (per one SD increase) | | **Hip size** (per one SD increase) | | **BMI** (per one SD increase) | |
|---|---|---|---|---|---|---|
| | HR (95% CI) | p-value | HR (95% CI) | p-value | HR (95% CI) | p-value |
| **Overall** (n=9011) | | | | | | |
| Traditional | **1.055 (1.031-1.080)** | **<0.001** | **1.069 (1.045-1.093)** | **<0.001** | **1.073 (1.048-1.097)** | **<0.001** |
| Residuals | 0.985 (0.963-1.008) | 0.197 | 1.013 (0.991-1.035) | 0.257 | | |
| Allometric | 0.984 (0.961-1.007) | 0.162 | 1.013 (0.990-1.036) | 0.276 | | |
| **Pre-MP** at baseline (n=2178) | | | | | | |
| Traditional | 0.976 (0.926-1.028) | 0.361 | 0.994 (0.947-1.044) | 0.823 | 0.977 (0.930-1.027) | 0.367 |
| Residuals | 0.991 (0.943-1.041) | 0.715 | 1.029 (0.983-1.077) | 0.221 | | |
| Allometric | 0.988 (0.941-1.038) | 0.643 | 1.028 (0.982-1.075) | 0.239 | | |
| **Post-MP** at baseline (n=5268) | | | | | | |
| Traditional | **1.083 (1.051-1.116)** | **<0.001** | **1.107 (1.075-1.139)** | **<0.001** | **1.116 (1.083-1.149)** | **<0.001** |
| Residuals | 0.972 (0.944-1.000) | 0.053 | 1.013 (0.985-1.043) | 0.361 | | |
| Allometric | 0.971 (0.942-1.000) | 0.051 | 1.011 (0.981-1.041) | 0.490 | | |
| **Diagnosis at age<55** years (n=1968) | | | | | | |
| Traditional | 0.973 (0.921-1.028) | 0.329 | 0.980 (0.931-1.032) | 0.448 | 0.964 (0.914-1.016) | 0.171 |
| Residuals | 1.010 (0.959-1.064) | 0.704 | 1.024 (0.976-1.075) | 0.330 | | |
| Allometric | 1.006 (0.956-1.059) | 0.817 | 1.026 (0.979-1.076) | 0.285 | | |
| **Diagnosis at age≥55** years (n=7043) | | | | | | |
| Traditional | **1.076 (1.049-1.104)** | **<0.001** | **1.093 (1.066-1.121)** | **<0.001** | **1.102 (1.075-1.131)** | **<0.001** |
| Residuals | 0.977 (0.952-1.002) | 0.068 | 1.008 (0.983-1.033) | 0.533 | | |
| Allometric | 0.976 (0.951-1.002) | 0.067 | 1.006 (0.981-1.032) | 0.630 | | |
| **ER+PR+** subtypes (n=3101) | | | | | | |
| Traditional | **1.090 (1.048-1.134)** | **<0.001** | **1.114 (1.072-1.157)** | **<0.001** | **1.120 (1.079-1.164)** | **<0.001** |
| Residuals | 0.972 (0.935-1.011) | 0.154 | 1.015 (0.978-1.054) | 0.430 | | |
| Allometric | 0.971 (0.933-1.010) | 0.141 | 1.016 (0.978-1.056) | 0.409 | | |
| **ER+PR-** subtypes (n=726) | | | | | | |
| Traditional | 0.935 (0.858-1.019) | 0.123 | 0.983 (0.904-1.068) | 0.682 | 0.994 (0.913-1.081) | 0.885 |
| Residuals | **0.898 (0.827-0.974)** | **0.010** | 0.986 (0.911-1.066) | 0.722 | | |
| Allometric | **0.894 (0.822-0.971)** | **0.008** | 0.981 (0.905-1.063) | 0.644 | | |
| **ER-PR-** subtypes (n=759) | | | | | | |
| Traditional | 0.941 (0.866-1.023) | 0.152 | 1.003 (0.926-1.086) | 0.945 | 0.992 (0.914-1.076) | 0.838 |
| Residuals | **0.911 (0.840-0.987)** | **0.022** | 1.031 (0.955-1.113) | 0.439 | | |
| Allometric | **0.906 (0.835-0.983)** | **0.018** | 1.028 (0.951-1.112) | 0.487 | | |
| **ER+/-PR-** subtypes (n=1485) | | | | | | |
| Traditional | **0.938 (0.883-0.996)** | **0.036** | 0.993 (0.938-1.052) | 0.823 | 0.993 (0.936-1.053) | 0.807 |
| Residuals | **0.904 (0.854-0.958)** | **<0.001** | 1.009 (0.955-1.066) | 0.739 | | |
| Allometric | **0.900 (0.849-0.954)** | **<0.001** | 1.006 (0.951-1.064) | 0.842 | | |

**ABSI** – a body shape index; **BMI** – body mass index; **CI** – confidence interval; **ER+/-** – oestrogen receptor status; **HI** – hip index; **HR** – hazard ratio; **n** – number of cases; **p-value** – from Wald test for the individual term; **Pre-MP** – pre-menopausal at baseline (the time of the anthropometric

assessment); **Post-MP** – post-menopausal at baseline; **PR+/-** – progesterone receptor status; **SD** – standard deviation.

Models included the following exposures on a continuous scale (z-scores, value minus mean divided by SD):

**Allometric** – a model including ABSI (mean 72.939; SD 5.070), HI (mean 64.806; SD 2.717), and BMI (mean 25.304; SD 4.464) as exposures;

**Residuals** – a model including as exposures BMI and residuals of waist and hip circumferences derived individually for each of them from linear regression on weight and height (mean 0 for both; SD 5.641 for waist residuals; SD 4.246 for hip residuals);

**Traditional** – a model including either waist circumference (mean 79.762; SD 11.162), or hip circumference (mean 100.694; SD 9.096), or BMI as exposures, each in a separate model.

**HR (95% CI)** (per one SD increase) were derived from Cox proportional hazards models, stratified by age at the anthropometric assessment, country, and categories of menopausal status and, for post-menopausal women, age at menopause (pre-menopausal, peri-menopausal, menopause at <46 years, menopause at 46 to <52 years, menopause at ≥52 years, menopause at unknown age), and adjusted for height (continuous), smoking status and intensity (never smoker, former quit≥15 years, former quit<15 years, current ≤10 cigarettes/day, current >10 cigarettes/day), alcohol consumption (none, <4 g/day, 4 to <16 g/day, ≥16 g/day), physical activity (inactive, moderately inactive, moderately active, active), education (primary/none, technical, secondary, university/longer), hormone replacement therapy use (never, former, current, missing), oral contraceptives use (never, former, current), age at the first period (continuous), parity with age at first live birth (nulliparous, one at <25 years, one at ≥25 years, two at <25 years, two at ≥25 years, ≥3 at <25 years, ≥3 at ≥25 years, missing), breastfeeding with duration (never, <6 months, ≥6 months, missing), and energy intake (log-transformed continuous).

**Supplementary Table S6 Associations of allometric anthropometric indices with breast cancer risk (sensitivity analyses)**

| | | **Main analysis** | | **Minimally adjusted** | | | **Follow-up ≥2 years** | | | **ER+/-PR+/-** | |
|---|---|---|---|---|---|---|---|---|---|---|---|
| | Cases | HR (95% CI) | p-value | HR (95% CI) | p-value | Cases | HR (95% CI) | p-value | Cases | HR (95% CI) | p-value |
| **ABSI** (per one SD increase) | | | | | | | | | | | |
| Overall | 9011 | 0.984 (0.961-1.007) | 0.162 | 0.981 (0.959-1.004) | 0.098 | 8023 | 0.976 (0.952-1.000) | 0.052 | 4692 | **0.948 (0.918-0.980)** | **0.001** |
| MP at baseline | | | | | | | | | | | |
| Pre-MP | 2178 | 0.988 (0.941-1.038) | 0.643 | 0.989 (0.942-1.038) | 0.657 | 1969 | 0.977 (0.928-1.029) | 0.372 | 1322 | 0.952 (0.894-1.014) | 0.125 |
| Post-MP | 5268 | 0.971 (0.942-1.000) | 0.051 | **0.965 (0.937-0.994)** | **0.018** | 4668 | **0.961 (0.931-0.992)** | 0.014 | 2606 | **0.928 (0.889-0.969)** | **<0.001** |
| $p_{heterogeneity}$ | | 0.535 | | 0.396 | | | 0.595 | | | 0.524 | |
| Age at diagnosis | | | | | | | | | | | |
| <55 years | 1968 | 1.006 (0.956-1.059) | 0.817 | 1.003 (0.954-1.055) | 0.912 | 1542 | 0.998 (0.942-1.057) | 0.938 | 1038 | 0.982 (0.915-1.054) | 0.611 |
| ≥55 years | 7043 | 0.976 (0.951-1.002) | 0.067 | **0.973 (0.948-0.999)** | **0.039** | 6015 | **0.970 (0.943-0.997)** | **0.033** | 3654 | **0.937 (0.903-0.971)** | **<0.001** |
| $p_{heterogeneity}$ | | 0.299 | | 0.300 | | | 0.387 | | | 0.247 | |
| ERPR status | | | | | | | | | | | |
| ER+PR+ | 3101 | 0.971 (0.933-1.010) | 0.141 | 0.964 (0.927-1.003) | 0.067 | 2853 | 0.970 (0.931-1.011) | 0.147 | | | |
| ER+PR- | 726 | **0.894 (0.822-0.971)** | **0.008** | **0.884 (0.814-0.961)** | **0.004** | 658 | **0.898 (0.823-0.980)** | **0.016** | | | |
| ER-PR- | 759 | **0.906 (0.835-0.983)** | **0.018** | **0.903 (0.833-0.978)** | **0.013** | 690 | **0.903 (0.829-0.983)** | **0.019** | | | |
| $p_{heterogeneity}$ | | 0.108 | | 0.100 | | | 0.143 | | | | |
| ER+/-PR- | 1485 | **0.900 (0.849-0.954)** | **<0.001** | **0.894 (0.844-0.947)** | <0.001 | 1348 | **0.901 (0.847-0.957)** | **<0.001** | | | |
| $p_{ER+/-PR- \text{ vs } ER+PR+}$ | | 0.037 | | 0.034 | | | 0.049 | | | | |
| **HI** (per one SD increase) | | | | | | | | | | | |
| Overall | 9011 | 1.013 (0.990-1.036) | 0.276 | 1.009 (0.986-1.031) | 0.452 | 8023 | 1.007 (0.983-1.031) | 0.586 | 4692 | 1.007 (0.976-1.039) | 0.644 |
| MP at baseline | | | | | | | | | | | |
| Pre-MP | 2178 | 1.028 (0.982-1.075) | 0.239 | 1.021 (0.976-1.068) | 0.364 | 1969 | 1.027 (0.979-1.077) | 0.277 | 1322 | 0.994 (0.938-1.053) | 0.832 |
| Post-MP | 5268 | 1.011 (0.981-1.041) | 0.49 | 1.007 (0.977-1.037) | 0.662 | 4668 | 1.002 (0.971-1.034) | 0.902 | 2606 | 1.015 (0.973-1.059) | 0.477 |
| $p_{heterogeneity}$ | | 0.543 | | 0.602 | | | 0.400 | | | 0.554 | |
| Age at diagnosis | | | | | | | | | | | |
| <55 years | 1968 | 1.026 (0.979-1.076) | 0.285 | 1.019 (0.972-1.069) | 0.426 | 1542 | 1.027 (0.973-1.084) | 0.337 | 1038 | 1.031 (0.966-1.100) | 0.359 |
| ≥55 years | 7043 | 1.006 (0.981-1.032) | 0.630 | 1.003 (0.978-1.029) | 0.802 | 6015 | 0.993 (0.966-1.021) | 0.628 | 3654 | 0.998 (0.964-1.035) | 0.933 |
| $p_{heterogeneity}$ | | 0.476 | | 0.561 | | | 0.282 | | | 0.398 | |

| | Main analysis | | | Minimally adjusted | | Follow-up ≥2 years | | | ER+/-PR+/- | | |
|---|---|---|---|---|---|---|---|---|---|---|---|
| | Cases | HR (95% CI) | p-value | HR (95% CI) | p-value | Cases | HR (95% CI) | p | Cases | HR (95% CI) | p-value |
| **HI** (per one SD increase) | | | | | | | | | | | |
| ERPR status | | | | | | | | | | | |
| ER+PR+ | 3101 | 1.016 (0.978-1.056) | 0.409 | 1.019 (0.981-1.059) | 0.333 | 2853 | 1.011 (0.972-1.053) | 0.575 | | | |
| ER+PR- | 726 | 0.981 (0.905-1.063) | 0.644 | 0.980 (0.905-1.061) | 0.617 | 658 | 0.983 (0.904-1.070) | 0.699 | | | |
| ER-PR- | 759 | 1.028 (0.951-1.112) | 0.487 | 1.026 (0.949-1.109) | 0.520 | 690 | 1.017 (0.937-1.103) | 0.695 | | | |
| $p_{\text{heterogeneity}}$ | | 0.682 | | 0.652 | | | 0.821 | | | | |
| ER+/-PR- | 1485 | 1.006 (0.951-1.064) | 0.842 | 1.003 (0.949-1.061) | 0.910 | 1348 | 1.001 (0.944-1.062) | 0.975 | | | |
| $p_{\text{ER+/-PR- vs ER+PR+}}$ | | 0.763 | | 0.651 | | | 0.773 | | | | |
| **BMI** (per one SD increase) | | | | | | | | | | | |
| Overall | 9011 | **1.074 (1.049-1.098)** | **<0.001** | **1.051 (1.028-1.074)** | **<0.001** | 8023 | **1.080 (1.055-1.107)** | **<0.001** | 4692 | **1.074 (1.041-1.109)** | **<0.001** |
| MP at baseline | | | | | | | | | | | |
| Pre-MP | 2178 | 0.980 (0.932-1.030) | 0.422 | 0.962 (0.917-1.010) | 0.116 | 1969 | 0.980 (0.930-1.033) | 0.459 | 1322 | 0.996 (0.935-1.061) | 0.895 |
| Post-MP | 5268 | **1.117 (1.085-1.150)** | **<0.001** | **1.087 (1.057-1.118)** | **<0.001** | 4668 | **1.130 (1.095-1.165)** | **<0.001** | 2606 | **1.124 (1.078-1.171)** | **<0.001** |
| $p_{\text{heterogeneity}}$ | | <0.001 | | <0.001 | | | <0.001 | | | 0.002 | |
| Age at diagnosis | | | | | | | | | | | |
| <55 years | 1968 | 0.966 (0.917-1.019) | 0.206 | **0.942 (0.895-0.991)** | **0.021** | 1542 | 0.957 (0.901-1.017) | 0.156 | 1038 | **0.910 (0.844-0.981)** | **0.014** |
| ≥55 years | 7043 | **1.104 (1.076-1.132)** | **<0.001** | **1.080 (1.054-1.107)** | **<0.001** | 6015 | **1.118 (1.088-1.149)** | **<0.001** | 3654 | **1.120 (1.081-1.160)** | **<0.001** |
| $p_{\text{heterogeneity}}$ | | <0.001 | | <0.001 | | | <0.001 | | | <0.001 | |
| ERPR status | | | | | | | | | | | |
| ER+PR+ | 3101 | **1.122 (1.080-1.165)** | **<0.001** | **1.093 (1.054-1.134)** | **<0.001** | 2853 | **1.145 (1.101-1.191)** | **<0.001** | | | |
| ER+PR- | 726 | 0.998 (0.917-1.086) | 0.956 | 0.957 (0.881-1.039) | 0.294 | 658 | 1.003 (0.918-1.096) | 0.947 | | | |
| ER-PR- | 759 | 0.996 (0.919-1.081) | 0.933 | 0.991 (0.917-1.072) | 0.824 | 690 | 1.002 (0.920-1.091) | 0.959 | | | |
| $p_{\text{heterogeneity}}$ | | 0.004 | | 0.003 | | | 0.002 | | | | |
| ER+/-PR- | 1485 | 0.997 (0.940-1.057) | 0.924 | 0.975 (0.921-1.031) | 0.374 | 1348 | 1.003 (0.943-1.066) | 0.929 | | | |
| $p_{\text{ER+/-PR- vs ER+PR+}}$ | | <0.001 | | <0.001 | | | <0.001 | | | | |

**ABSI** – a body shape index; **CI** – confidence interval; **BMI** – body mass index; **ER+/-** – oestrogen receptor status; **HI** – hip index; **HR** – hazard ratio; **p-value** – from Wald test for the individual term; **Pre-MP** – pre-menopausal at baseline (the time of the anthropometric assessment); **Post-MP** – post-menopausal at baseline; **PR+/-** – progesterone receptor status; **SD** – standard deviation.

**HR (95% CI)** (per one SD increase) were derived from Cox proportional hazards models including ABSI, HI, and BMI on a continuous scale as exposures (z-scores, value minus mean (72.939 for ABSI; 64.806 for HI; 25.304 for BMI) divided by SD (5.070 for ABSI; 2.717 for HI; 4.464 for BMI)), stratified by age at the anthropometric assessment, country, and categories of menopausal status and, for post-menopausal women, age at menopause (pre-menopausal, peri-menopausal, menopause at <46 years, menopause at 46 to <52 years, menopause at ≥52 years, menopause at unknown age);

**Main model** – a stratified model adjusted for height (continuous), smoking status and intensity (never smoker, former quit≥15 years, former quit<15 years, current ≤10 cigarettes/day, current >10 cigarettes/day), alcohol consumption (none, <4 g/day, 4 to <16 g/day, ≥16 g/day), physical activity (inactive, moderately inactive, moderately active, active), education (primary/none, technical, secondary, university/longer), hormone replacement therapy use (never, former, current, missing), oral contraceptives use (never, former, current), age at the first period (continuous), parity with age at first live birth (nulliparous, one at <25 years, one at ≥25 years, two at <25 years, two at ≥25 years, ≥3 at <25 years, ≥3 at ≥25 years, missing), breastfeeding with duration (never, <6 months, ≥6 months, missing), and energy intake (log-transformed continuous);

**Minimally adjusted** – a stratified model adjusted only for height (continuous);

**Follow-up≥2 years** – fully adjusted stratified model excluding women with less than two years of breast cancer follow-up;

**ER+/-PR+/-** – fully adjusted stratified model censoring breast cancers with unknown hormone receptor status at the date of diagnosis;

**$p_{\text{heterogeneity}}$**; **$p_{\text{ER+/-PR- vs ER+PR+}}$** – derived with the data augmentation method of Lunn and McNeil [21].
